# Macrophages Contribute to the Cyclic Activation of Adult Hair Follicle Stem Cells

**DOI:** 10.1371/journal.pbio.1002002

**Published:** 2014-12-23

**Authors:** Donatello Castellana, Ralf Paus, Mirna Perez-Moreno

**Affiliations:** 1 Epithelial Cell Biology Group, BBVA Foundation-CNIO Cancer Cell Biology Programme, Spanish National Cancer Research Centre (CNIO), Madrid, Spain; 2 Institute of Inflammation and Repair, University of Manchester, Manchester, United Kingdom; 3 Department of Dermatology, University of Münster, Münster, Germany; Stanford University School of Medicine, Howard Hughes Medical Institute, United States of America

## Abstract

Skin epithelial stem cells operate within a complex signaling milieu that orchestrates their lifetime regenerative properties. The question of whether and how immune cells impact on these stem cells within their niche is not well understood. Here we show that skin-resident macrophages decrease in number because of apoptosis before the onset of epithelial hair follicle stem cell activation during the murine hair cycle. This process is linked to distinct gene expression, including Wnt transcription. Interestingly, by mimicking this event through the selective induction of macrophage apoptosis in early telogen, we identify a novel involvement of macrophages in stem cell activation *in vivo*. Importantly, the macrophage-specific pharmacological inhibition of Wnt production delays hair follicle growth. Thus, perifollicular macrophages contribute to the activation of skin epithelial stem cells as a novel, additional cue that regulates their regenerative activity. This finding may have translational implications for skin repair, inflammatory skin diseases and cancer.

## Abstract 2

Castellana, Paus, and Perez-Moreno discover that skin resident macrophages signal to skin stem cells via Wnt ligands to activate the hair follicle life cycle.

## Author Summary

The cyclic life of hair follicles consists of recurring phases of growth, decay, and rest. Previous studies have identified signals that prompt a new phase of hair growth through the activation of resting hair follicle stem cells (HF-SCs). In addition to these signals, recent findings have shown that cues arising from the neighboring skin environment, in which hair follicles dwell, also participate in controlling hair follicle growth. Here we show that skin resident macrophages surround and signal to resting HF-SCs, regulating their entry into a new phase of hair follicle growth. This process involves the death and activation of a fraction of resident macrophages— resulting in Wnt ligand release —that in turn activate HF-SCs. These findings reveal additional mechanisms controlling endogenous stem cell pools that are likely to be relevant for modulating stem cell regenerative capabilities. The results provide new insights that may have implications for the development of technologies with potential applications in regeneration, aging, and cancer.

## Introduction

Epithelial homeostasis relies on the capability of epithelium to self-renew over a lifetime because of the presence of diverse reservoirs of stem cells (SCs). These reside in anatomically distinct niches that provide them with a specialized microenvironment, which are becoming increasingly well-defined in the largest and most accessible mammalian organ, the skin [1]. Besides its epithelial components, the skin contains both resident and migratory immune cell populations, whose major role is mainly attributed to its function as a central line of defense for fighting infection, as well as promoting skin repair upon injury and external assaults [2]. During wound repair, coordinated and carefully balanced crosstalk between epithelial and inflammatory cells occurs to restore skin homeostasis [2]–[4]. Failure in this communication is associated with major wound healing defects, inflammatory disorders, and malignant transformation [5],[6].

The exact functional relationship of specific immune cell populations in the activation of epithelial progenitor cells in adult mammalian skin is, however, still poorly defined. Moreover, how resident immunocytes interact with epithelial SCs *in vivo* is not fully understood. Such interactions can be optimally studied in the best-characterized reservoir of adult skin epithelial SCs, the hair follicle (HF) bulge [7],[8].

The bulge is located around the level of insertion of the arrector pili muscle into the HF epithelium below the sebaceous gland, enjoys a relative immune privilege [9]–[11], and is ensheathed by a specialized mesenchyme, the connective tissue sheath (CTS) [12]–[14], which is richly endowed with macrophages and mast cells that home into this skin compartment early during HF development [15]. Bulge SCs (HF-SCs) are the essential prerequisite for the cyclic regeneration of HFs, during which it switches from phases of growth (anagen) via regression (catagen) to relative quiescence (telogen) [7],[16]. HF entry into anagen requires the activation of HF-SCs and of progenitors located in the secondary hair germ (HG) that expand to give rise to a new anagen HF [17]–[19].

Important for the activation of HF-SCs at the end of telogen is the close and dynamic interaction with a specialized condensate of inductive fibroblasts, the dermal papilla (dp), which provides a specialized microenvironment [14]. Recently, other intercellular interactions within the HF niche and with its mesenchymal environment have become appreciated as key elements of HF-SC activation [12],[13]. These elements include signals in the niche itself that arise from the HF-SC progeny [20], and signals of the tissue macroenvironment arising from dermal fibroblasts, adipocytes [21] and preadipocytes [22], and nerve fibers [23]. However, despite their prominence in the HF mesenchyme, including in the peri-bulge CTS [15], the role of perifollicular macrophages in HF-associated epithelial-mesenchymal interactions has remained unclear.

Recent studies have contributed greatly to our understanding of the key role of two major signaling pathways in the intrinsic activation of HF-SCs and the entry of HF into anagen. These pathways are the stimulatory Wnt/β-catenin signaling pathway [24],[25], and the inhibitory bone morphogenetic protein (BMP) signals arising from the dp that uphold HF-SCs in a quiescent state [24],[25]. Interestingly, these signals are also exploited by the skin macroenvironment, which generates synchronized cyclic waves of BMP activity that decline when Wnt expression waves arise, thereby controlling HF cycling. These cyclic waves respectively subdivide telogen into refractory and competent phases for HF regeneration [21]. Remarkably, HF growth stimulatory signals can also be propagated during the transition from telogen to anagen via neighboring HFs [26]. Whether immune cells located in the perifollicular macroenviroment, such as macrophages, contribute to the establishment of the refractory and competent phases of telogen, or in the propagation of the HF growth stimulatory cues is much less clear.

It is now firmly established that mature HFs have a distinctive immune system [11],[27]. Indeed, both the HF bulb and the HF bulge represent areas of immune privilege [9],[11],[28], whose collapse gives rise to distinct inflammatory hair loss disorders [10],[29]. Interestingly, HFs are constantly in close interaction with immune cells, namely intraepithelially located T lymphocytes and Langerhans cells, and macrophages and mast cells located in the HF's CTS [15],[30]–[32]. The HF epithelium also may serve as portal for the entry of immune cells into the epidermis, such as dendritic cells [33], as a habitat for both fully functional and immature Langerhans cells [34] and as a potent source of chemokines that regulate dendritic cell trafficking in the skin [33].

Prior studies have shown that intracutaneous immune cell populations fluctuate substantially in number and activities during synchronized HF cycling [27],[33],[35]–[41]. While it is known that this fluctuation results in major changes in skin immune responses (e.g., inhibition of contact hypersensitivity in anagen skin [35]), and in the intracutaneous signaling milieu for various immunomodulatory cytokines and chemokines [33],[42], it is insufficiently understood whether these hair cycle-associated changes are a consequence of HF cycling or if they actively regulate the latter and/or the hair cycle-associated activity of HF-SCs.

For example, perifollicular mast cells and macrophages have been implicated in the regulation of HF growth through anagen and the entry into catagen [15],[36]–[41],[43]. Namely, timed release of the catagen-inducing growth factor, Fgf5, by perifollicular macrophages may regulate the anagen-catagen switch [36],[44], while clustering of macrophages around isolated HFs may serve to delete selected pilosebaceous units [30]. Most recently, it has been shown that loss of γδT cells, which are required for HF neogenesis induced upon wounding [4], results in hair cycling abnormalities [42].

Whereas these studies have implicated immune cells in HF cycling, their role in the spatio-temporal cyclic activation of HF-SCs, specifically in the physiological entry of telogen HFs into anagen, remains to be defined. Using the murine hair cycle as a model system and focusing on macrophages, we have addressed this important, as yet uncharted aspect of HF-immunocyte interactions. These studies define a new role for skin-resident macrophages in the activation of HF-SCs.

## Results

### Skin-Resident Myeloid Cells Decrease in Number as Telogen Advances to Anagen

**Figure 1:**
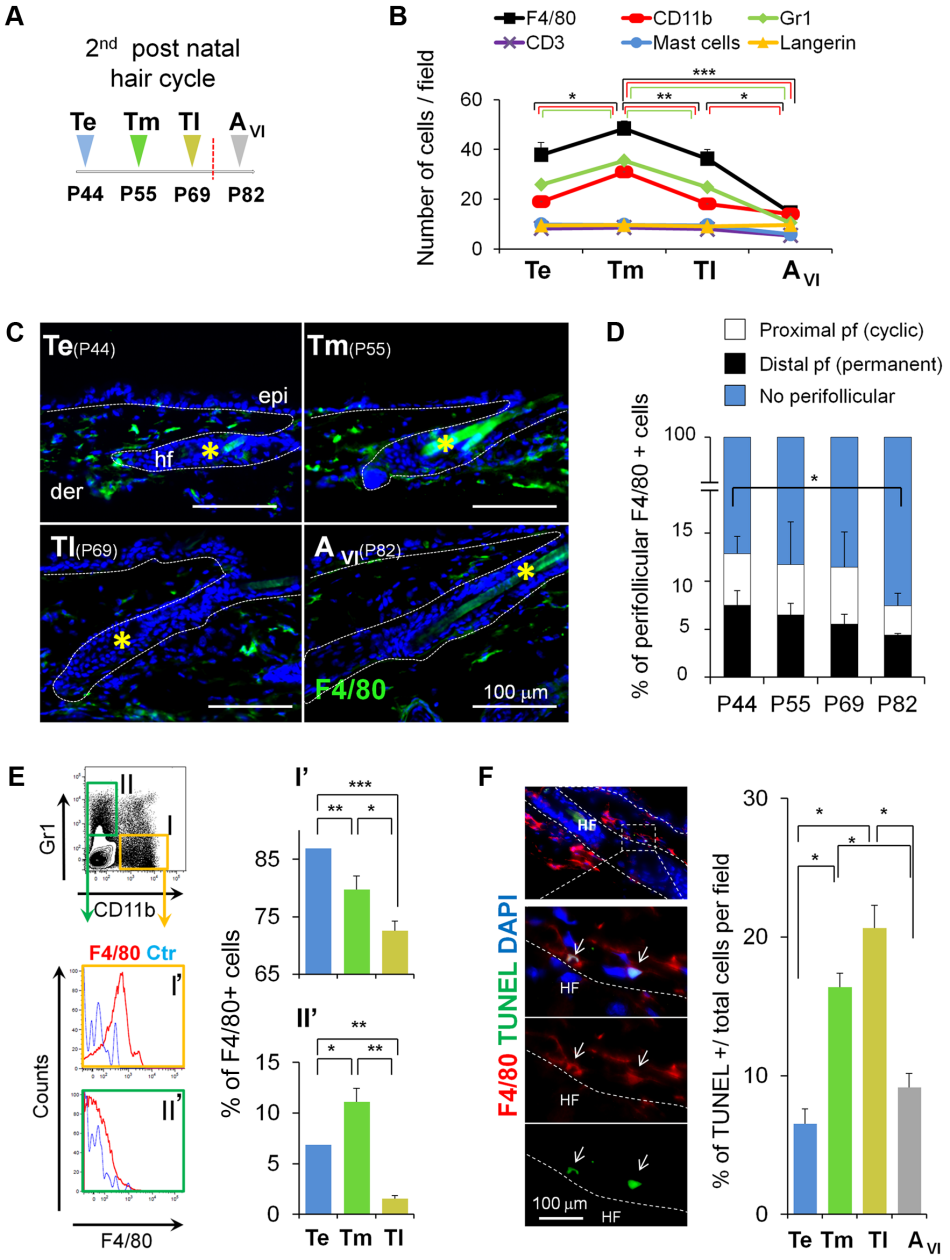
Skin-resident macrophages decrease in number before the onset of anagen. (A) Time course of isolation of backskin samples. The second telogen period was subdivided into three stages followed by anagen. P44, early telogen (Te); P55, mid telogen (Tm); P69, late telogen (Tl); and P82, anagen (A_VI_). (B) Fluctuations in the number of skin-resident immune cells during the second hair cycle analyzed by immunofluorescence. Note the decrease in cells expressing the myeloid markers CD11b, Gr1, F4/80 before the onset of anagen. Each histogram point represents the mean value of positive cells per 10× magnification field. 10 fields/section/mouse were analyzed; *n* = 4. See also Figure S1. (C) Expression of F4/80 cells (green) in skin at Te, Tm, Tl, and A_VI_ stages, counterstained with DAPI (blue). Bar = 100 µm; *hair shaft autofluorescence. (D) Fluctuations in the number of perifollicular macrophages during the second hair cycle analyzed by immunofluorescence. Note the decrease in F4/80^+^ at A_VI_. Each histogram point represents the mean value of positive cells per 10× magnification field at 30 µm distance from HF [15]. 10 fields/section/mouse were analyzed; *n* = 4. (E) FACS analysis of single cell suspensions from total skin samples harvested at Te, Tm, and Tl stages. Histograms show the percentage of F4/80^+^ cells gated from the CD11b^+^Gr1^−^cells (I) and Cd11b^−^Gr1^+^ (II) populations; *n* = 7–12. The gating strategy is shown in Figure S3A. (F) TUNEL^+^F4/80^+^ cells in Tm. Histograms show the percentage of TUNEL positive cells in the FACS sorted CD11b^+^F4/80^+^Gr1^−^ macrophage population (I) analyzed in cytospin preparations; *n* = 3. The gating strategy is shown in Figure S11 A. Note: *n* refers to the number of mice, per point per condition. *p≤0.05; **p<0.005; ***p<0.0005. All data used to generate the histograms can be found in Data S1.

To evaluate the association of HF-SC activation with specific populations of skin-resident inflammatory cells, we first performed immunofluorescence analyses in mouse backskin sections isolated from matched areas of defined phases of spontaneous murine HF cycling. These analyses were performed from the telogen through the anagen phase of the first (Figure S1A), and the second postnatal hair cycle (Figure 1A). The telogen phase of the first HF cycle lasts only for 1–2 days, whereas the second telogen starts around postnatal day 44 (P44) and last for 3–4 weeks. Thus, we subdivided the second telogen in three telogen stages, the early telogen stage (Te, Postnatal day 44, P44), mid telogen (Tm, P55), late telogen (Tl, P69), and included an anagen stage (A_VI_, P82) according to the classification of Muller Rover [45], to perform our comparative analyses (Figure 1A). The second telogen corresponded to the refractory and competent telogen phases [21], as supported by the analysis of BMPs and Wnts transcript levels (Figure S2).

We observed that the number of Langerhans cells (Langerin), mast cells (toluidine blue), and T-lymphocytes (CD3) were not significantly different in these stages (Figures 1B, S1C, and S1D). However, the number of myeloid cells (F4/80, CD11b, and Gr1) increased at Tm and progressively decreased at Tl before the onset of HF-SC activation as observed by immunofluorescence (Figure 1B and 1C) and fluorescence-activated cell sorting (FACS) analyses (Figure S3). This global decrease was observed in the dermis (no perifollicular) but also in macrophages located near the distal (close to the epidermis) and proximal portion of HFs as Te progresses to anagen (Figure 1D and 1E).

Moreover, analyses of skin whole mount stainings and 3-D reconstructions showed that ∼50% of HFs in telogen exhibited F4/80^+^ cells, and only 10% of HFs displayed dense perifollicular inflammatory cell clusters (PICCs) as previously defined (Figure S4) [30]. Interestingly, in the short transition from telogen to anagen of the first postnatal HF cycle, a decrease in F4/80 and CD11b, but not in Gr1 positive cells was also observed (Figure S1B). We also confirmed that through the first anagen phase (from A_IIIa_ to A_VI_) there was an increase in the numbers of these cells, consistent with previous reports [15],[36]. Since different populations of macrophages reside in skin, we performed flow cytometry (FACS) analyses in total skin samples during the second telogen (Figure 1A) to obtain a more detailed analysis of their phenotype and number.

To analyze the number of F4/80^+^ cells, mature resident macrophages were gated from either CD11b^+^ or Gr1^+^ (Ly6G^+^) cells (Figures 1E and S3A). This allowed us to differentiate CD11b^+^Gr1^−^F4/80^+^ macrophages (I′), from the myeloid CD11b^−^Gr1^+^F4/80^+^ population (II′). These analyses showed that CD11b^+^F4/80^+^Gr1^−^ macrophages gradually decreased from Te to Tl, whereas CD11b^−^F4/80^+^Gr1^+^ myeloid cells increased in number at Tm, followed by a significant decrease at Tl (Figure 1E). Of note, no changes were observed in either the number of CD11c^+^ cells in total murine skin, or of F4/80^+^ cells present within this dendritic cell population (Figure S3B).

Next, we asked whether the observed numeric reduction of macrophages towards the end of telogen and before anagen induction (Figure 1B and 1E) was due to macrophage apoptosis. TUNEL analyses of skin sections co-stained with F4/80 revealed the presence of F4/80^+^/TUNEL^+^ cells at HF distal, proximal, and no perifollicular regions (Figures 1F and S1F). In addition, TUNEL analyses in FACS-isolated CD11b^+^Gr1^−^F4/80^+^ cells from total skin showed a significant increase in apoptosis, when isolated from skin that progressed from Tm to Tl (Figure 1F), consistent to the subG1 peak observed in their cell cycle profile (Figure S1G). Taken together, these data suggest that the telogen-anagen switch of the hair cycle is associated with an apoptosis-driven reduction of skin-resident macrophages.

### Experimental Ablation of Skin-Resident Macrophages Induces Precocious HF Entry into Anagen

Our results raised the intriguing hypothesis that the observed decrease in mature skin resident macrophages may be related to HF-SC activation and anagen induction. To probe this possibility and characterize the relevance of macrophages in the activation HF-SCs, we attempt to use inducible LysMCre-diptheria toxin receptor (DTR) mice, which express DTR in myeloid cells [46]. After DT administration, myeloid cells are susceptible to ablation. However, although this model is well-characterized under conditions of wound repair [47], we did not observe the expression of LysM^+^ resident cells in skin using the reporter mice LysMCre-Katushka under steady state conditions, as compared to the expression in the bone marrow derived macrophages (BMDMs), liver, and spleen (Figure S5). This observation may be explained by the fact that at least two different lineages of macrophages exist in mice, one derived from hematopoietic SCs, and the other derived from the yolk sac closely associated with epithelial structures [48]. Thus, we turned to chemical targeting via clodronate-induced macrophage apoptosis [49] in early telogen skin, to mimic the reduction in macrophage numbers. We focused on the second HF cycle, which is routinely exploited in hair research to dissect hair cycle-regulatory signals [18],[24],[50]–[52]. We performed subcutaneous injections of clodronate-encapsulated liposomes (CL-lipo), which are specifically engulfed by macrophages and induce their apoptosis [49],[53]. Because of its selectivity, this cell ablation system is widely used to explore the role of macrophages in other systems [54]–[56].

**Figure 2:**
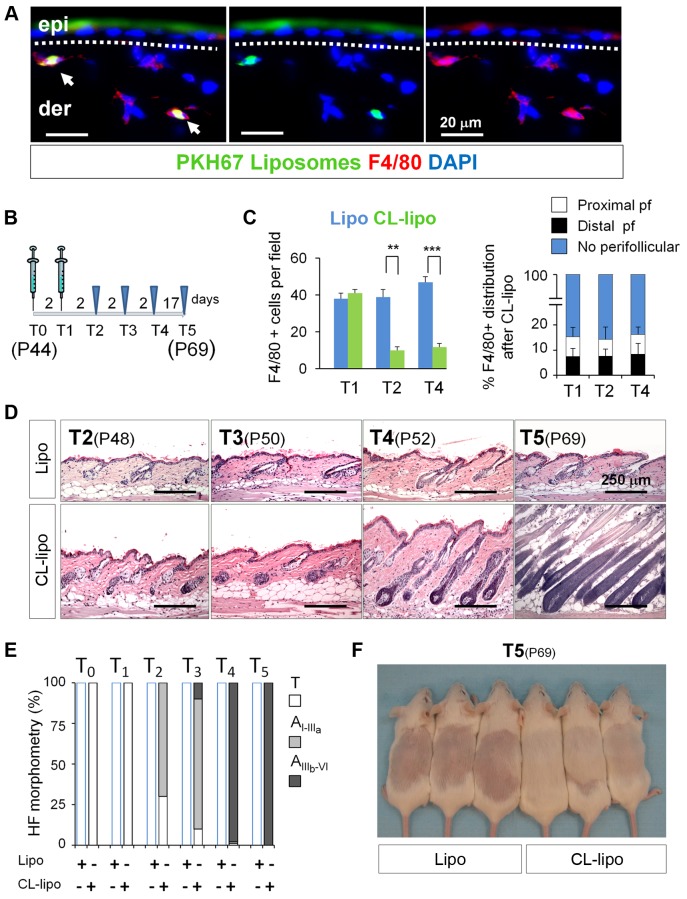
Reduction of macrophage numbers in early telogen induces precocious HF growth. (A) The specific uptake of liposomes by macrophages was analyzed by co-immunofluorescence analysis of F4/80^+^ cells (red) and the detection of the liposomal PKH67 label (green) in skin sections after the injection of PKH67-liposomes. Arrows indicate double labeling; *n* = 2. Bar = 20 µm. (B) P44 mice were injected in the backskin for two alternated days with CL-lipo. Samples were collected for analyses at the time indicated in the diagram. (C) Histograms represent the quantification of F4/80^+^ cells in the backskin after treatment with CL-lipo and Lipo (left). Also shown is the distribution of F4/80^+^ cells in the backskin after treatment with CL-lipo (right). 10 fields/section/mouse were analyzed; *n* = 4. (D) Hematoxylin–eosin staining of backskin samples isolated after treatment with CL-lipo and Lipo controls. Bar = 250 µm, *n* = 4. (E) Histomorphometric analysis of HF stages after macrophages reduction. 100 HFs/mouse were analyzed; *n* = 4. (F) Appearance of the hair coat at T5 (P69), after shaving and treatment with CL-lipo and Lipo controls at T0 (P44). **p<0.005; ***p<0.0005. Note: *n* refers to the number of mice, per point per condition. All data used to generate the histograms can be found in Data S1.

First, empty PKH67-labeled liposomes were subcutaneously injected as controls, and backskins from matched areas were collected to avoid HF regional differences in skin [21],[52]. The specific uptake of the injected PKH67-liposomes by skin-resident macrophages was confirmed by double immunofluorescence analyses of PKH67 labeled membranes and F4/80 (Figures 2A and S6A). Next, we examined the effectiveness of the treatment at different time points after its administration (Figure 2B), and observed that F4/80^+^ cell numbers in skin were significantly reduced at T2 and T4 at HF distal, proximal, and no-perifollicular regions (Figures 2C and S6B). TUNEL analyses showed an increase in F4/80^+^ apoptotic cells starting from T1 (Figure S6C). This reduction was also observed for CD11b^+^ and Gr1^+^ cells (Figure S6D). Overall, the final number of resident macrophages was similar to the one at physiological Tm and Tl stages (Figure 1B).

We then assessed the effect of experimentally decreasing macrophage numbers at Te on hair growth. Strikingly, histological analyses revealed that as soon as macrophage levels were reduced (T2), HF entered into anagen (Figure 2D). At T4, while HFs in control animals were still in telogen (P52), nearly 100% of the HFs of CL-lipo-treated mice entered into anagen, as shown by quantitative hair cycle histomorphometry (Figure 2E). These differences were phenotypically noticeable by the premature appearance of the hair coat in the previously shaved backskin of CL-lipo-treated mice, when compared with controls (T5) (Figure 2F). Of note, the observed anagen-promoting effects of macrophage reduction in HF growth does not seem to be strain specific, since it can also be observed in another mouse strain in the areas of CL-lipo injection (Figure S6E and S6F).

**Figure 3:**
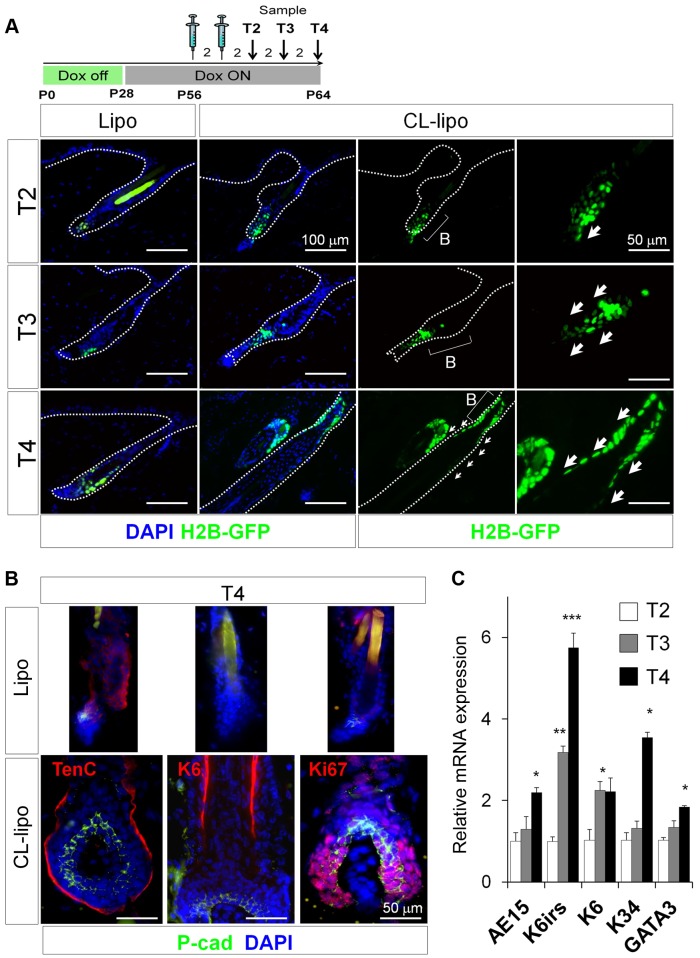
Reduction of macrophage numbers in Telogen induces precocious exiting and differentiation of HF-SCs. (A) Representative backskin sections of K5tTA-pTREH2B-GFP mice, subjected to a pulse-chase treatment with doxycycline, followed by treatment at P56 for two alternated days with CL-lipo or Lipo controls. Arrows show the mobilization of LRCs (green); *n* = 3. Dox, doxycycline. (B) Immunofluorescence analysis of P-cad, Tenascin C (TenC), Keratin6 (K6), and the proliferation marker Ki67; *n* = 4. *Hair shaft autofluorescence. Bar = 50 µm. (C) Relative mRNA expression of the differentiation markers trichohyalin (AE15), K6irs, K6, K34, and GATA3 from total backskin samples after treatment with CL-lipo, normalized to Lipo controls; *n* = 2–4. *p≤0.05. All data used to generate the histograms can be found in Data S1.

Next we analyzed the effect of experimentally decreasing macrophage numbers on bulge HF-SCs, which are characterized by their slow cycling properties (label retaining cells [LRCs]) [17],[57], whereas their progeny divides rapidly to expand and migrate [18],[19] giving rise to the matrix progenitor cells and the generation of fully mature HFs [18],[19],[58]. To this end, we performed pulse-chase strategies using doxycycline-regulated keratin 5 (K5)tTA (TetOff)-Histone H2B-GFP mice [17]. After finishing the chase at P56, we treated the mice for two alternate days with CL-lipo and observed a proportion of LRCs outside the bulge when compared to controls (Figures 3A and S7A).

Moreover, the precocious entry of HFs into anagen occurred with no obvious alterations in HF differentiation. Immunostaining analyses confirmed the presence of Ki67^+^ proliferative cells in the hair matrix along with the expression of P-cadherin (P-cad), as well as the distribution of the companion layer marker keratin 6 (K6) and the extracellular matrix protein tenascin C (TenC), all in the expected HF locations (Figure 3B). The expression of K6irs, K34, GATA3, and the inner root sheet marker trichohyalin (AE15) was also analyzed in total skin at mRNA level (Figure 3C). Globally, these data suggest that the reduction of macrophages during telogen induces a precocious exiting and differentiation of HF-SCs.

### CL-lipo Treatment Is Macrophage-Specific and Induces Neither HF Toxicity Nor Skin Inflammation

As CL-lipo-induced toxicity and inflammation might have generated this effect, we systematically probed this possibility. Since the rate of intraepithelial HF apoptosis is a very sensitive indicator of HF damage (dystrophy) [59],[60], it is important to note no major signs of apoptosis were observed in epithelial cells compared to controls (Figure S6G).

Furthermore, no changes were observed in the number of other immune cells, including T-cells (CD3), mast cells, and B-cells (Pax5) in skin (Figure S6D), supporting that CL-lipo treatment was macrophage-selective. This was further corroborated by the observation that subcutaneous CL-lipo treatment did not impinge on the number of monocytes and macrophages of bone marrow, spleen, or peripheral blood (Figure S7B and S7C).

This finding was in line with the observation that CL-lipo induced neither an increase in the expression of the prototypic pro- and anti-inflammatory cytokines, interleukin-10 (IL10) and -12 (IL12), respectively, in skin (Figure S7D). In addition, no changes in the expression of the proinflammatory molecule ICAM1 were observed in skin, even after 2 and 4 d post treatment (T2 and T3) (Figure S7E). However, ICAM1 of the HF epithelium increased at late stages upon CL-lipo treatment (T4), consistent with the documented upregulation of ICAM-1 expression in anagen_VI_ HFs just before their entry into catagen [61].

### Reduction of Skin Macrophages Is Associated with Activation of β-catenin/Wnt Signaling

**Figure 4:**
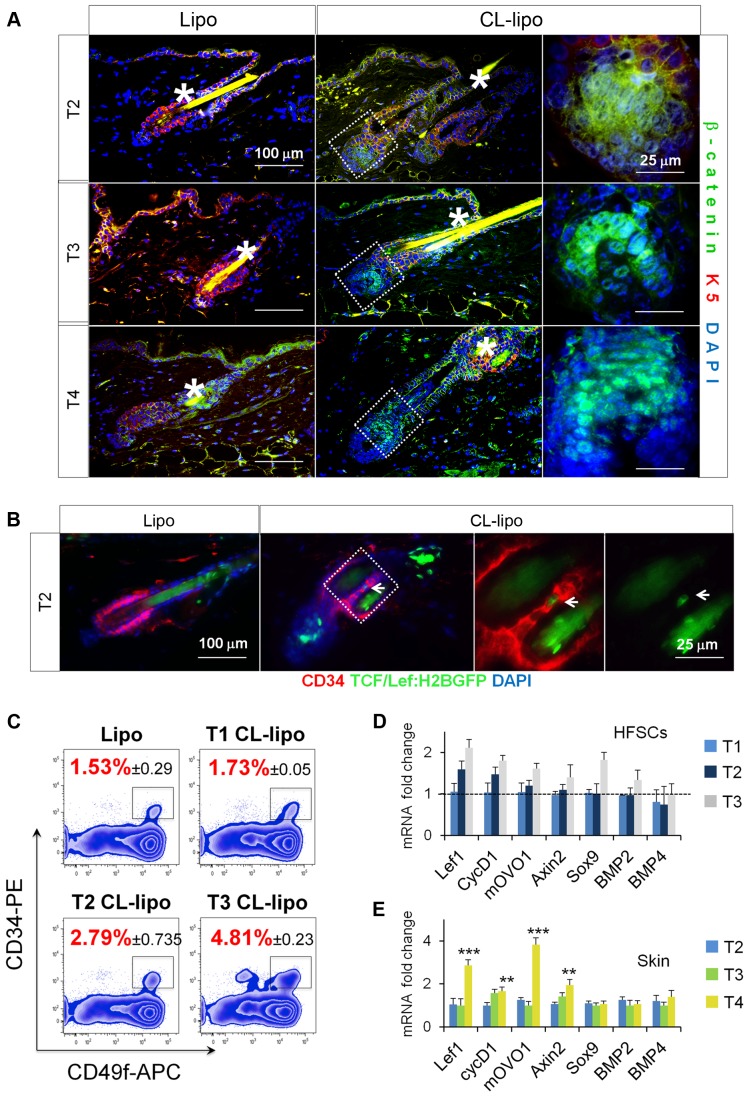
Reduction of skin macrophages is associated with activation of β-catenin/Wnt signaling. (A) Immunofluorescence analysis of β-catenin (green) and K5 (red) in backskin sections of mice treated with CL-lipo and Lipo controls; *n* = 4. *Hair shaft autofluorescence. Bar = 25 µm. (B) Immunofluorescence analysis of CD34 (red) and H2B-GFP signal (green) in T2 backskin sections of TCF/Lef:H2B-GFP transgenic mice treated with CL-lipo and Lipo controls; *n* = 3. Arrows point to GFP positive cells. Bar = 25 µm. The gating strategy is shown in Figure S11 B. (C) FACS analysis of single cell suspensions of CD34^+^CD49f^+^ HF-SCs (gated) isolated from backskin of mice treated with CL-lipo or Lipo controls at specified time points; *n* = 2–4. (D) Relative mRNA expression of canonical Wnt/β-catenin target genes and BMP signaling genes in HF-SCs isolated as indicated in (B); *n* = 2–4. (E) Relative mRNA expression of canonical Wnt/β-catenin target genes and BMP signaling genes in total back-skin samples after treatment with CL-lipo compared to Lipo controls; *n* = 6. Note: *n* refers to the number of mice, per point per condition. *p≤0.05. All data used to generate the histograms can be found in Data S1.

Due to the recognized fundamental role of Wnt/β-catenin signaling in HF-SC activation and HF growth [24],[62]–[66], we next analyzed the distribution of β-catenin after CL-lipo treatment by immunofluorescence. Interestingly, nuclear β-catenin was detected in HFs early after CL-lipo treatment (T2) (Figure 4A). In addition, under the background of TCF/Lef:H2B-GFP transgenic mice [67], the CL-lipo treatment induced signs of H2B-GFP expression in few CD34^+^ bulge cells and in the HG at T2, not observed in Lipo controls (Figure 4B). This level of activation is consistent with physiological levels as previously documented [68]. We also performed RT-PCR analyses in FACS-isolated HF-SCs (Figure 4C) and observed an increase in their number and in the relative mRNA expression levels of the Wnt signaling related genes Lef1 [68],[69], and mOVO1 [70] and Axin2 [71] starting from T2, without any changes in the expression levels of the HF-inhibitory proteins BMP2 and BMP4 (Figure 4D) [18],[21],[25],[72]. As expected these increases were also observed in total skin at late stages of anagen when the matrix forms and HFs differentiate (Figure 4E). These data support an association between macrophages and the β-catenin/Wnt signaling in the activation of HF-SCs.

### Resident Macrophages Express HF-SC Stimulatory Factors before the Onset of Anagen

**Figure 5:**
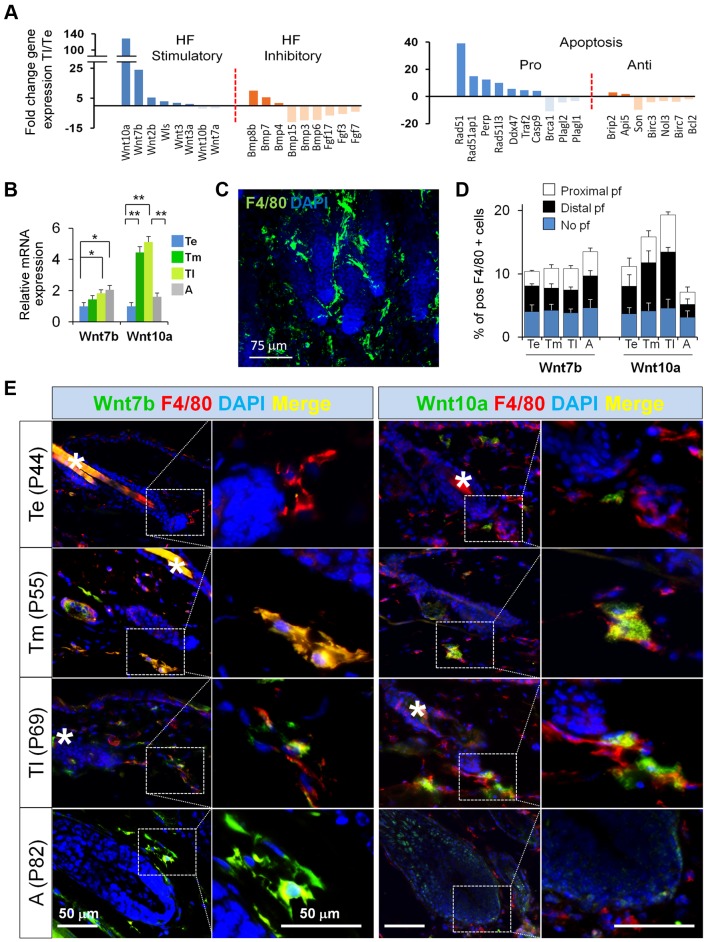
Resident macrophages express HF-SC stimulatory factors before the onset of anagen. (A) CD11b^+^Gr1^−^F4/80^+^ macrophages were FACS-isolated from Te and Tl backskin samples. Their mRNAs were purified and used to perform microarray analyses to evaluate changes in gene expression at Tl (P69) versus Te (P44). Histograms show a shortlist of up- and downregulated genes that have been involved in the control of HF-SC activation and apoptosis. The gating strategy is shown in Figure S3 A. (B) Relative mRNA expression of Wnt7b and Wnt10a in FACS-sorted CD11b^+^Gr1^−^F4/80^+^ cells at Te, Tm, Tl, and A; *n* = 3. The gating strategy is shown in Figure S3A. (C) Immunofluorescence staining of F4/80^+^ perifollicular macrophages (green). (D) Each histogram point represents the mean value of double positive F4/80^+^Wnt7b^+^ and F480^+^Wnt10a^+^ over total F4/80^+^ perifollicular macrophages. 10 fields/section/mouse were analyzed; *n* = 4. (E) Immunofluorescence of Wnt7b (green)/F4/80 (red), and Wnt10a (green)/F4/80 (red), counterstained with DAPI (blue) of skin sections at Te, Tm, Tl, and A; *n* = 3. Bar = 50 µm. n.s. non significant, Note: *n* refers to the number of mice, per point per condition. *p≤0.05. All data used to generate the histograms can be found in Data S1.

To obtain mechanistic insight into how macrophages control the activation of HF-SCs under physiological steady-state conditions, we performed microarray analysis of the CD11b^+^Gr1^−^F4/80^+^ skin resident macrophages at physiological Te, Tm, and Tl in order to characterize changes in their gene expression profile as HFs progress from telogen to anagen. Figures 5A and S8A show the results of the comparison between late and early telogen (Tl/Te).

Interestingly, genes involved in the regulation of HF-SC behavior were found to be the most upregulated ones in macrophages before the onset of HF-SC activation, among them Wnt7b and Wnt10a ligands that can activate canonical β-catenin/Wnt signaling. Moreover, the expression of pro-apoptotic genes was higher at Tl when compared to Te, consistent with the observed increase in macrophage apoptosis (Figures 1F, S1F, and S1G), correlating apoptosis with the expression of Wnts.

In addition, we confirmed that skin resident macrophages are highly heterogeneous. Indeed, immunofluorescence analysis revealed that some macrophages coexpressed markers of both M1/M2 phenotypes, such as iNOS (M1) and Arg1 (M2), under these uninflamed conditions (Figure S8B–S8D). In total skin, no changes were detected in the mRNA expression of cytokines such as IL10 and IL12a, two key cytokines that are important for the alternative and inflammatory properties of macrophages, respectively (Figure S8E).

We next validated the increase in the expression levels of Wnt7b and Wnt10a preceding the onset of anagen. We first performed quantitative reverse transcription (RT)-PCR assays in FACS-sorted macrophages isolated from physiological Te, Tm, Tl, and anagen stages. Consistent with the microarray data, the mRNA expression levels of both Wnt7b and Wnt10a increased as HF transitioned from Te to A (Figure 5B). This increase appeared to reflect primarily expression changes within macrophages, since Gr1^+^ cells did not display any changes in Wnt7b and Wnt10a expression (Figure S8F). Wnt7b mRNA levels were maintained at the beginning of anagen, while Wnt10a levels decreased to ∼50% (Figure 5B).

Interestingly, immunofluorescence analyses revealed the presence of clusters of perifollicular macrophages (Figures 5C and S4), reminiscent of PICCs [30], and during the progression of telogen these exhibited both Wnt7b and Wnt10a expression in close proximity to the HFs and less pronounced in the no perifollicular zone (Figure 5D and 5E). Although technical limitations in obtaining sufficient macrophage numbers precluded the biochemical analysis of Wnt7b and Wnt10a protein levels in macrophages during these stages, these results demonstrate an intriguing association between macrophage-derived Wnt expression and HF-SC activation.

### Macrophage Apoptosis Is Associated with an Increase of Wnt7b and Wnt10a

To investigate whether both Wnt7b and Wnt10a can be produced autonomously by macrophages, we turned to *in vitro* studies. As expected, the *in vitro* treatment of BMDM with CL-lipo was able to stimulate apoptosis in a large fraction of macrophages (∼35%). Most interestingly, this resulted in the release of cell-accumulated Wnt7b and Wnt10a into the media (BMDM conditioned media [CM]) (Figure S9A–S9C).

To further assess the effect of apoptosis on the expression and release of Wnts, we cultured BMDM derived from the LysMCre^+/T^ iDTR^KI/KI^ mice, or control BMDM^KI/KI^ (Figure S9D). DT treatment triggered the apoptosis of LysMCre^+/T^ iDTR^KI/KI^ BMDM, but not control cells (Figure S9E). Surviving cells, apoptotic cells, and their respective supernatants were collected and analyzed by immunoblot. This showed that Wnt7b protein levels in cell lysates slightly increased in apoptotic LysMCre^+/T^ iDTR^KI/KI^ BMDM (Figure S9E). However, both Wnts were increased in the CM when compared to controls (Figure S9F).

We then stimulated fresh control BMDM cells with the previously described surviving (LysMCre^+/+^ iDTR^KI/KI^ BMDM), apoptotic cells (LysMCre^+/T^ iDTR^KI/KI^ BMDM), or their respective CM. The stimulation of fresh BMDM with apoptotic BMDM upregulated the expression of Wnt10a (Figure S9G), whereas no effect in the expression of Wnts was observed upon stimulation with their CM (Figure S9G).

Overall, these murine macrophage cell culture data suggest that macrophage apoptosis goes along with the release of Wnts and that close intercellular interactions between macrophages are important for apoptotic macrophages to further stimulate the expression of Wnts of neighboring macrophages.

### Apoptotic Macrophages Can Activate HF-SCs *In Vitro*

Next, we probed the causal association of macrophages with HF-SC activation *in vitro* by assessing the effect of the BMDM CM in cultured HF-SC. To this end, we FACS-isolated CD34^+^K15-GFP^+^ cells from the backskin of K15-GFP mice (Figure S10A) [73]. HF-SCs were cultured and stimulated with CM of BMDM treated with CL-lipo or control liposomes (Lipo) (Figure S10A). Consistent with the *in vivo* data reported above (Figure 4), treatment of HF-SCs with media conditioned by CL-lipo BMDM significantly and reproducibly induced the expression of canonical Wnt downstream targets in HF-SCs, including CycD1, Lef1, and axin2 (Figure S10B).

As control for specificity, we treated HF-SCs directly with CL-lipo or Lipo, and no phagocytic uptake was observed by HF-SCs, neither changes in the expression of the analyzed transcripts (Figure S10B). In addition, immunofluorescence studies revealed the expression of K1 and K10 differentiation markers, without an increase in Ki67^+^ cells when compared to controls (Figure S10C), in agreement with previous data indicating the capacity of HF-SCs to differentiate into epidermal lineages *in vitro* [74]. Overall, these findings suggest that macrophages contribute to the activation of HF-SCs.

### Inhibition of the Production of Wnts by Skin Macrophages Delays Anagen

To investigate the involvement of macrophage-derived Wnts in the activation of HF-SCs and anagen induction under physiological conditions, we subcutaneously injected liposomes containing the specific hydrophobic small molecule inhibitor of Wnts, IWP-2. IWP-2 is a bona-fide broad Wnt inhibitor that specifically prevents palmitoylation of Wnt proteins, thereby blocking Wnt their processing and activity [75]–[77]. It was to our great advantage that this inhibitor is embedded and retained in the liposome membrane. As shown in Figure 2A, the delivery and uptake of liposomes selectively occurs in phagocytic macrophages. Moreover, IWP2-liposomes have been successfully used to block Wnt activity derived from macrophages in other systems [78].

**Figure 6:**
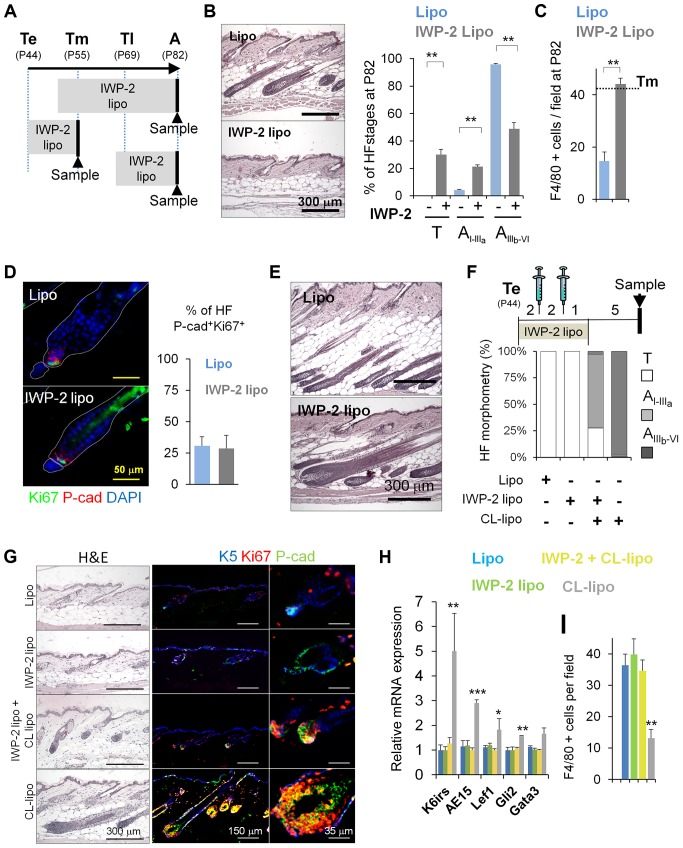
Inhibition of the production of Wnts by skin macrophages delays hair growth. (A) Scheme illustrating the subcutaneous treatment with IWP-2-liposomes (IWP-2-lipo) to target mature phagocytic macrophages at different telogenic stages. Arrowheads indicate the time in which skin samples were collected. (B) Histological analyses of skin sections harvested at A (P82), after being treated with IWP-2-lipo starting before Tm (P50); *n* = 8. Quantification of the stage of HFs telogen (T), Early anagen (A_I–IIIa_), late anagen (A_IIIb–VI_); *n* = 8. (C) Histogram shows the quantification of the number of F4/80+ cells at A (P82), after being treated with IWP-2-lipo starting before Tm (P50). 10 fields/section/mouse were analyzed; *n* = 3. Dotted line represents Tm threshold levels. (D) Mice were injected in the backskin with IWP-2-lipo and Lipo controls starting at Te (P44). Samples were collected at Tm (P55), and processed for immunofluorescence analyses of Ki67 and P-cad (HG). The histogram shows the percentage of HFs with a HG positive for Ki67^+^Pcad^+^ cells; *n* = 3. (E) Histological analysis of skin sections harvested at A (P82) after being treated with IWP-2-lipo starting at P67, two days before Tl; *n* = 3. (F–G) Histomorphometric analysis of HF stages and histological analyses of skin sections of mice treated for 5 days with IWP2-lipo and Lipo controls. Over the curse of this treatment, CL-lipo and Lipo controls (syringes) were administered twice every 2 days. Samples were collected 6 days later. Immunostaining shows proliferating HG (Ki67^+^P-cad^+^). 100 HFs/mouse were analyzed; *n* = 6. (H) Relative mRNA expression of HF differentiation markers in total skin samples treated as described in (F); *n* = 6. (I) Histogram shows the quantification of the number of F4/80^+^ cells per field, after being treated as described in Figure 6F; *n* = 3. Note: *n* refers to the number of mice, per point per condition. *p≤0.05, **p<0.005; ***p<0.0005. All data used to generate the histograms can be found in Data S1.

Using this approach, we performed treatments at different telogen stages (Figure 6A, Te, Tm, and Tl). Strikingly, the sustained inhibition of Wnts starting at Tm was sufficient to delay the HF-SC entry into anagen and prevented the reduction of macrophage numbers (Figure 6B and 6C). Of note, the treatment with IWP-2 liposomes at Te (Figure 6D) or at Tl (Figure 6E), did not have an effect in HF-SCs and HG proliferation, and HF growth when compared to controls. Overall, these results indicate that macrophages contribute to the activation of HF-SCs, leading to a permissive state that allows HF entry into anagen.

Inhibition of the processing of Wnts derived from macrophages via IWP2-liposomes dampened the anagen-inducing effect of CL-lipo treatment, as documented by histological and immunofluorescence analysis of P-cad (enriched in the HG) (Figure 6F and 6G), and by the quantitative mRNA expression of HF-differentiation markers in total skin (Figure 6H). Under these conditions, the treatment with IWP-2 liposomes also abrogated the reduction of macrophage numbers (Figure 6I).

Taken together, our results suggest that the apoptosis-associated secretion of Wnts by perifollicular macrophages contributes to the activation of epithelial HF-SCs, allowing HF entry into anagen.

## Discussion

While previous studies have already pointed to a link between macrophages and the regulation of HF cycling, in particular during the anagen-to-catagen transition [15],[30],[36], the current study provides the first evidence, to our knowledge, that a selective reduction in the number of macrophages induces premature anagen entry. Moreover, our data suggest that changes in the release of Wnt signals by perifollicular macrophages may contribute to the establishment of the refractory and competent phases of telogen, and to the propagation of cues that induce anagen. Finally, we show that apoptotic macrophages can activate epithelial HF-SCs in a Wnt-dependent manner, and that inhibition of Wnts derived from macrophages delays anagen.

Conceptually, this finding reveals that skin-resident macrophages function as important mesenchymal regulators of epithelial HF-SC function under physiological conditions and identifies a novel link between macrophages and HF cycling. Given, however, the many similarities between anagen development and wound healing on the one hand [79], and the key role of skin macrophages in wound repair on the other [47], it is not surprising that macrophages turn out to be involved not only in matrix scavenging during HF regression [43], but also in HF-SC activation and anagen induction. Thus, our study underscores the importance of macrophages as modulators of tissue regeneration and organ remodeling, well beyond their function as phagocytes, and highlights that the murine hair cycle offers an excellent model for further dissection of these physiological roles.

The fact that a reduction in skin macrophage numbers exerts strong hair cycle-modulatory effects corresponds to the previously reported hair cycle-accelerating effects of γδ T cell deletion [42], and points to the need for systematic re-examination of the role of immunocytes in hair growth control. This line of research should facilitate the development of novel therapeutic strategies for the manipulation of undesired human hair loss or growth that target perifollicular immunocytes, such as macrophages. Particularly important will be the studies focusing on human inflammatory permanent alopecias characterized by irreversible HF-SC damage and macrophage infiltration of the bulge [10].

We noted that ∼50% of the HFs of the second postnatal telogen exhibited perifollicular F4/80^+^ cells. Previous findings of a much smaller percentage of perifollicular macrophage clusters (PICCs) (∼2%) [30] likely reflect differences in the hair cycle stage analyzed (first postnatal anagen and during the transition of anagen-to-catagen) [30]. Furthermore, our analyses revealed that the number of macrophages declines as telogen progresses from the refractory to the competent phases of telogen (Figures 1 and S1), probably after performing their phagocytic functions during the basement membrane resorption of involuting catagen HFs [43]. This scenario seems to be different when growing anagen HFs progress to catagen, as previously reported during the first HF cycle [15],[36], and confirmed here (Figure S1B).

Future work in this field should strive to use genetic mouse models to selectively decrease skin macrophage numbers, rather than having to rely on the clodronate method. However this process is difficult, given the differential origins of macrophages [48],[80],[81]. Our results stress the need to analyze the characteristics of skin resident macrophages and their differential roles in homeostasis (fate-mapping studies, linear tracing) to generate useful genetic mouse models not available to date.

The macrophage expression profiles identified in our studies underscored the highly heterogeneous phenotype of skin macrophages [82]–[84]. In the context of M1 and M2 macrophages [82],[85], they seem to comprise unpolarized populations since they co-express both M1/M2 markers in uninflamed, not wounded conditions. However, a clear upregulation of the expression levels of Wnt7b and Wnt10a was observed in macrophages as telogen progresses to anagen. Intriguingly, our observation that apoptosis upregulates the expression of Wnts is fully consistent with observations documented in other systems, including e.g., Hydra and liver models [55],[86]. Wnt7b activity has been implicated in regenerative processes including macrophage-dependent control of cell fate decisions in the vasculature [87], lung development [88], and macrophage-dependent kidney wound repair [89]. Moreover, Wnt10a is upregulated during HF development [90], and Wnt10a missense mutations have been associated with the human syndromes odonto-onycho-dermal dysplasia [91] and Schöpf–Schulz–Passarge [92],[93], both characterized for malformations in ectodermal structures.

Macrophages have been extensively implicated in the development of several tissues, as well as in homeostasis and cancer [85],[94]–[96]. They have been directly implicated in the regulation of other adult SC niches such as the hematopoietic SCs [97],[98], mammary SCs [99], and liver [55]. However, macrophage functions have specific roles depending on the tissue context [94]. Hence, dissecting the roles of skin-resident macrophages in homeostatic HF regenerative conditions adds a new relevant facet of skin biology. It is an important first step in understanding the functions of macrophages in other contexts such as skin repair, skin inflammatory diseases, and cancer.

In skin repair, it has been recently documented that macrophages play differential roles as wounds heal [47]. Interestingly, their infiltration upon wounding is required for HF growth [100]. It is well-established that HF-SCs transiently contribute to the epidermal lineage after injury to support cutaneous wound healing [17],[101]–[103], and that large full thickness wounds induce HF neogenesis [4],[101]. Hence, future research should target the involvement of different skin epithelial progenitor cells, macrophages, and macrophage derived Wnts in these contexts. In addition, since adult skin HF-SCs, their immediate progeny, and basal progenitor cells have been identified as cells of origin of skin carcinomas [104],[105], the elucidation of HF-SC interactions with macrophage-derived Wnts in the context of tumorigenesis [85],[106] is an important question for future studies.

Our study delineates that macrophage-derived Wnts activate HF-SCs and HF entry into anagen. In addition, our results raise the possibility that non-apoptotic perifollicular macrophages operate as an “immunocyte brake” on HF-SC activation, which is only released by the macrophage apoptosis-associated release of Wnts. This finding begs the next question to be addressed in subsequent studies: What triggers and regulates perifollicular macrophage apoptosis during telogen? For example, does this numeric decline only reflect the natural completion of the finite macrophage life span, or does the HF epithelium (including its SCs) actively participate in the reduction of macrophages? Overall, we surmise that the outcome of HF-SC activation via macroenviromental signals is regulated by a whole host of tightly regulated signaling loops between HF-SCs, adipocytes, immune cells, the vasculature, and now, based on our findings, with macrophages.

Determining whether these molecular signals are orchestrated along with the intrinsic HF-SC regulatory cues will be valuable to define the multiple hierarchies that underlie HF regeneration. Once powerful tools of molecular biology at hand in mice become applicable to human hair research, including novel *in situ*-imaging tools to assess HF-SC activation in humans [107], new translationally and therapeutically relevant insights into the macrophage-epithelial SC connection and its role in tissue remodeling, organ repair, and hair diseases may be achievable.

## Materials and Methods

### Ethics Statement

All protocols related to animal research were approved by the Animal Experimental Ethics Committee of the Carlos III Health Institute, in strict compliance with institutional guidelines and the international regulations for Welfare of Laboratory Animals.

### Mice and Treatments

Experiments were performed with 6- to 12-week old Crl:CD1 (ICR) and FVB/N female mice. Mice were sacrificed at specific postnatal days (P), and their dorsal skins were dissected and processed for analyses. To reduce the number of skin-resident macrophages, 1 mg of clodronate-encapsulated liposomes were administered to mice via daily subcutaneous injections during two alternated days (Encapsula Nanosciences). CL-lipo are the one of the most effective, specific, and extensively used agents to deplete phagocytic monocytes and macrophages via apoptosis [49],[53]. The specific Wnt inhibitor IWP-2 (Roche Diagnostics) was encapsulated in liposomes (Encapsula Nanosciences) and 50 µg were injected subcutaneously [75],[78]. The K5 tTA(TetOff)-histone H2B-GFP mice [17], the K15-GFP mice (Jackson Lab) [103], the Katushka reporter mice [108], and the TCF/Lef:H2B-GFP transgenic mice (Jackson Lab) [67] have been previously described. Doxycycline treatments were initiated in 28 d postnatal mice [17], and maintained until the collection of samples after the performance of subcutaneous injections of CL-lipo and Lipo at specified times.

### Microarray Analysis

Total RNAs from FACS isolated skin-resident macrophages, pooled from three littermate mice per point, were purified using the Absolutely RNA reverse transcription system (Stratagene). These samples were provided to the CNIO Genomics Core Facility to perform the quantification, assessment of RNA quality, labeling, hybridization, and scanning process. Briefly, 0.05–1 ng RNA were subjected to a preliminary amplification step with a TransPlex Whole Transcriptome Amplification WTA2 kit (Sigma). 250 ng of sample were reverse transcribed using the Agilent Oligonucleotide Array-Based CGH for Genomic DNA Analysis - ULS Labeling for Blood, Cells, Tissues or FFPE (with a High Throughput option). The recommendations from Sigma for the integration of TransPlex WTA with the Agilent microarray workflow were followed, such as the omission of Cot-1 DNA. 250 ng of cDNA were non-enzymatically labeled with either Cy3 or Cy5 fluorophores using the ULS technology (Kreatech), and labeled samples were hybridized to the Mouse Gene Expression G3 8×60 K array (Agilent) at 65°C for 40 h. Hybridized chips were scanned using a G2505C DNA microarray scanner (Agilent) and the obtained images were quantified using the Feature Extraction Software 10.7 (Agilent). Probesets were considered as differentially expressed when the absolute fold change was ≥10-fold. Unsupervised clustering analysis (UPGMA) was performed using Pearson correlation. The microarray data from this publication have been submitted to the GEO database http://www.ncbi.nlm.nih.gov/geo/info/linking.html and assigned the identifier GSE58098.

### Flow Cytometry and Cell Sorting

Backskins were minced into small pieces and digested in PBS, 1% BSA, 0.5 mg/ml DNase I, and 0.5 mg/ml collagenase II and IV for 1 h at 37°C. Single cell-suspensions were obtained via pipette mechanical dissociation of total skin (epidermis and dermis) followed by filtration through 40 µm cell strainers. Cells were washed in PBS, blocked using the mouse seroblock FcR reagent (CD16/CD32; BD Pharmigen), and stained for FACS analysis in ice-cold PBS, 0.5% BSA, 0.3 mM EDTA using the following antibodies: CD11b-PerCPCy5.5 (rat mAb Clone M1/70, 45-0112 eBioscience), F4/80-APC-eFluor780 (rat mAb Clone BM8, 47-4801 eBioscience), Gr1-PECy7 (rat mAb Clone RB6-8C5, 25-5931 eBioscience). To isolate HF-SC, backskins from K15-GFP mice were digested with 0.25% trypsin-EDTA in PBS for 14 h at 4°C. Cell suspensions were processed as mentioned above, and stained for 30 min at 4°C using the following antibodies: CD34-PE (rat mAb Clone RAM34; BD Pharmigen), CD49f-APC (rat mAb Clone eBioGoH3; eBioscience), and P-cad-APC (rat mAb Clone 106020; R&D systems). Cells were sorted on a FACSAria Ilu using the CellQuest Pro software (BD Biosciences), or analyzed using a FACSCanto and the FlowJo software (TreeStar). For Sub-G1 analysis sorted cells were fixed in 70% EtOH and stained with propidium iodide (Becton Dickinson). For TUNEL analyses, cytospin preparations of FACS-sorted cells were processed and stained according to the in situ Cell Death Detection kit, Fluorescein (Roche). The FCS files from this publication have been deposited in the Dryad repository http://dx.doi.org/10.5061/dryad.2822t [109].

### Statistical Analysis

All quantitative data are presented as mean ± SEM. Results are representative of at least three independent experiments. To determine the significance of the data obtained for two groups, comparisons were made using two-tailed, unpaired Student's *t* test. For all statistical analysis a confidence level of *p*≤0.05 was considered to be statistically significant.

## Supporting Information

Figure S1 **Skin-resident macrophages decrease in number before the onset of the first anagen.** (A) Backskin samples were isolated from three different stages: P20, telogen (T); P23, early anagen (Ae), and P29, late anagen (Al). (B) Histograms show the fluctuations in number of different immune cell types analyzed by immunofluorescence. Each histogram point represents the mean value of positive cells per 10× magnification field. 10 fields/section/mouse were analyzed; *n* = 4. *p≤0.05. (C) Histological analysis of the expression of Toludine blue positive mast cells in the backskin at Te, Tm, Tl, and A stages. The boxed areas are shown at higher magnification in the right panels. (D) Immunofluorescence analysis of CD3 (green) counterstained with DAPI (blue) in the backskin at the specified HF stages; *p≤0.05. The boxed areas are shown at higher magnification in the right panels. (E) Diagram showing the perifollicular (proximal and distal) and no perifollicular regions used to assess the distribution of macrophages. (F) Histogram shows the percent of TUNEL^+^F4/80^+^ cells in mouse backskin at different stages; *n* = 3. The gating strategy is shown in Figure S3 A. (G) Histogram shows the percent of Sub-G1 DNA fragmentation of F4/80^+^CD11b^+^ sorted cells from skin at different stages; *n* = 3. The gating strategy is shown in Figure S11A. All data used to generate the histograms can be found in Data S1.
(TIF)

Figure S2 **BMP/Wnt mRNA fluctuation at different telogenic stages.** (A) Graphs represent the relative mRNA expression levels of BMPs and Wnts in total skin at different telogenic stages. (B) Graphical representation of the fluctuation of mRNA levels. All data used to generate the histograms can be found in Data S1.
(TIF)

Figure S3 **FACS analyses of macrophage populations in skin samples at different telogenic stages.** (A) Gating strategy of single cell suspensions of total skin. Cells were analyzed by FACS and sorted by the differential expression of F4/80 on CD11b^−^Gr1^+^ and CD11b^+^Gr1^−^ populations at different time points. (A′) Histograms represent the percent of single F4/80^+^,CD11b^+^,Gr1^+^ positive cells in the total skin at different telogenic stages. (B) Single cell suspensions were analyzed for the co-expression of CD11c and F4/80. (B′) Quantification of CD11c^+^ single cells and (B″) double positive CD11c^+^F4/80^+^ are shown in the histogram at different time points; *n* = 4. All data used to generate the histograms can be found in Data S1. The gating strategy is shown in Figure S11C.
(TIF)

Figure S4 **HFs exhibit perifollicular macrophages.** (A) Immunofluorescence staining for F4/80^+^ cells (red) in whole mount skin preparations shown at different telogenic stages. (B) The histogram represents the percent of HFs exhibiting perifollicular macrophages. 200 HFs/mouse; *n* = 3. (C) The histogram represents the percent of HFs exhibiting PICCs clusters at different telogenic stages. 200 HFs/mouse; *n* = 3. (D) 3-D whole mount reconstruction of skin showing the distribution of F4/80^+^ cells, obtained using the Imaris software. *p≤0.05. All data used to generate the histograms can be found in Data S1.
(TIF)

Figure S5 **Skin resident macrophages do not present LysM-dependent expression of Cre under steady state conditions.** Immunofluorescence analyses of backskin, cytospin of BMDM, liver and spleen derived from LysMCre^+/T^, iDTR^KI/KI^, and control LysMCre^+/+^, iDTR ^KI/KI^ mice under the background of the red fluorescent Katushka mice. F480+ (green), Katushka (red), DAPI (blue); *n* = 4 mice.
(TIF)

Figure S6 **Subcutaneous administration of clodronate liposomes does not alter the number of other inflammatory cells in skin, and also is able to induce precocious HF growth at early telogen in FVB/N mice.** (A) The specific uptake of liposomes by macrophages was analyzed by co-immunofluorescence analysis of F4/80^+^ cells (red) and the detection of the liposomal PKH67 label (green) in skin sections after the injection of PKH67-liposomes. Arrows indicate double labeling; *n* = 2. (B) Immunofluorescence of F4/80+ in backskin section of mice treated with CL-lipo and Lipo controls and collected at different time points; *n* = 4. (C) Left. Histogram shows the percent and the distribution of TUNEL^+^F4/80^+^ cells in the backskin of mice treated with CL-lipo and Lipo and analyzed at different time points; *n* = 3. Right. TUNEL and F4/80 immunofluorescence analyses in T2 backskin samples of mice treated with CL-lipo; *n* = 3. (D) Histogram shows the number of inflammatory cells present in skin sections after treatment with CL-lipo and Lipo controls, detected by immunofluorescence or histology techniques; *n* = 4. (E) FVB/N mice were injected in the backskin at T0 for two alternated days with CL-lipo. Samples were collected for analyses at T4 (P52). Hematoxylin–eosin staining of backskin samples isolated after treatment with CL-lipo and Lipo controls. Bar = 250 µm; *n* = 2. (F) Appearance of the hair coat at T5 (P69) in FVB/N mice, after shaving and treatment with CL-lipo and Lipo controls at T0 (P44). Bar = 250 µm; *n* = 2. (G) TUNEL and K5 immunofluorescence analyses in T2 backskin samples of mice treated with CL-lipo; *n* = 3. All data used to generate the histograms can be found in Data S1.
(TIF)

Figure S7 **Subcutaneous administration of clodronate liposomes does not alter macrophage number in the spleen, blood, and bone marrow, nor does it induce skin inflammation.** (A) Representative backskin sections of K5tTA-pTREH2B-GFP mice, subjected to a pulse-chase treatment with doxycycline, followed by treatment at P56 for two alternated days with CL-lipo or Lipo controls. (B) Histograms show the number of F4/80, CD11b, and Gr1 positive cells in the bone marrow and peripheral blood detected by FACS, after subcutaneous treatment with CL-lipo and Lipo controls; *n* = 3. The gating strategy is shown in Figure S11D and S11E. (C) Histograms show the number of F4/80, CD11b, and Gr1 positive cells in the spleen detected by IF, after subcutaneous treatment with CL-lipo and Lipo controls; *n* = 3. (D) Histograms represent the relative mRNA expression levels of IL10 and IL12 at T4 in Lipo versus CL-lipo treated backskin; *n* = 3. (E) Histograms represent the relative ICAM1 mRNA expression levels at T2, T3, and T4 in Lipo versus CL-lipo treated backskin, and untreated Te and A_VI_; *n* = 3. *p≤0.05. All data used to generate the histograms can be found in Data S1.
(TIF)

Figure S8 **Macrophage gene expression during the Te to Tl transition.** (A) Unsupervised clustering heat map showing genes up- and downregulated in F4/80^+^CD11b^+^ cells isolated from backskin of mice at Te and Tl. (B) Histograms show a shortlist of classical or alternative macrophage associated genes that were found up- or downregulated in FACS-isolated CD11b^+^Gr1^−^F4/80^+^ mature macrophages, using microarray analyses. The gating strategy is shown in Figure S3A. (C) Immunofluorescence analysis of skin sections of iNOS (M1), Arg1 (M2), and F4/80. (D) Histograms represent the percentage of Arg1/iNOS double positive F4/80 cells in skin at different telogenic stages. (E) Histograms show the relative mRNA expression of IL10 and IL12a in whole backskin of mice and time point indicated; *n* = 3. (F) Relative mRNA expression of Wnt7b and Wnt10a in FACS-isolated F4/80^+^ cells present in the CD11b^−^Gr1+ population at Te, Tm, and Tl; *n* = 3. The gating strategy is shown in Figure S3 A. All data used to generate the histograms can be found in Data S1.
(TIF)

Figure S9 **Treatment of bone marrow differentiated macrophages with clodronate-liposomes releases Wnt7b and Wnt10a.** (A) Scheme illustrating the treatment of BMDM with either CL-lipo or Lipo controls, before harvesting treated cells or their conditioned medium (BMDM CM). (B) FACS analysis of the sub-G1 DNA content of BMDM treated with CL-lipo and Lipo controls; *n* = 3. Bars indicate the percentage of cell death. The gating strategy is shown in Figure S11 F. (C) Immunoblot analysis of Wnt7b and Wnt10a expression in both BMDM total cell lysates and BMDM CM treated with CL-lipo and Lipo controls. (D) Scheme illustrating the experimental approach used to explore the effect of apoptosis in the expression of Wnts. BMDM derived from LysMCre^+/T^-iDTR^KI/KI^ mice or control LysMCre^+/+^iDTR^KI/KI^ were treated with diphteria toxin (DT). Floating apoptotic (LysMCre^+/T^-iDTR^KI/KI^+DT) and alive attached (LysMCre^+/+^iDTR^KI/KI^+DT) macrophages were collected, and used to treat control BMDM in a 1∶1 ratio. (E) Immunoblot analysis of Wnt7b and Wnt10a and active caspase-3 (AC3) expression in BMDM and CM isolated from both LysMCre^+/T^-iDTR^KI/KI^ and control LysMCre^+/+^iDTR^KI/KI^ mice treated with DT; *n* = 3. (F) Immunoblot analysis of Wnt7b and Wnt10a expression in BMDM and CM isolated from LysMCre^+/+^iDTR^KI/KI^ mice treated with BMDM LysMCre^+/T^-iDTR^KI/KI^ and control LysMCre^+/+^iDTR^KI/KI^ mice treated with DT; *n* = 3. *n* refers to number of experimental replicates. (G) Immunoblot analysis of Wnt7b and Wnt10a expression in fresh BMDM treated with surviving LysMCre^+/+^iDTR^KI/KI^ cells or apoptotic LysMCre^+/T^-iDTR^KI/KI^ cells, or with their respective CM; *n* = 3. *n* refers to number of experimental replicates. All data used to generate the histograms can be found in .
(TIF)

Figure S10 **Macrophage derived soluble factors promote** ***in vitro*** **HF-SC activation and differentiation.** (**A**) Scheme representing the protocol used to stimulate HF-SCs with macrophage CM. BMDM cells were treated with either CL-lipo or Lipo controls. The media was collected and used to treat FACS-isolated GFP^+^, CD34^+^ HF-SCs growing in culture. The gating strategy is shown in Figure S11G. (**B**) Relative mRNA expression of HF-SCs treated with Lipo control, CL-lipo, or the BMDM CM of cells treated with Lipo and CL-lipo; *n* = 9. *n* refers to number of experimental replicates. (**C**) Immunofluorescence analysis of K1, K10, and Ki67 (red) in HF-SCs treated with CL-lipo BMDM CM when compared to controls. The histogram shows the quantification of positive cells; *n* = 3. *p≤0.05; ***p<0.0005. All data used to generate the histograms can be found in Data S1.
(TIF)

Figure S11 **Gating strategy of the flow cytometry analyses presented in this study.** The gating strategy is presented in Figures 1F, 4B, S1G, S3B, S7B, S9B, and S10A.
(TIF)

Data S1 **Data used to generate histograms in this study.** The table relates to Figures 1–6, S1, S2, S3, S4, and S6, S7, S8, S9, S10.
(XLSX)

Text S1 **Supplementary materials and methods.**
(DOC)

## Abbreviations

A: anagen
BMDM: bone marrow derived macrophage
BMP: bone morphogenetic protein
CL-lipo: clodronate-encapsulated liposomes
CM: conditioned media
CTS: connective tissue sheath
DTR: diptheria toxin receptor
FACS: fluorescence-activated cell sorting
HF-SC: bulge stem cell
HF: hair follicle
HG: hair germ
IL: interleukin
K: Keratin
Lipo: control liposomes
LRC: label retaining cell
P-cad: P-cadherin
PICC: perifollicular inflammatory cell cluster
SC: stem cell
Te: early telogen stage (postnatal day 44, P44)
Tl: late telogen (Tl, P69)
Tm: mid telogen (Tm, P55)
