# Supplementary figures and images for: Macrophages Contribute to the Cyclic Activation of Adult Hair Follicle Stem Cells
Source: PLoS Biol. 2014 Dec 23;12(12):e1002002. doi: 10.1371/journal.pbio.1002002 (PMC4275176; doi:10.1371/journal.pbio.1002002)

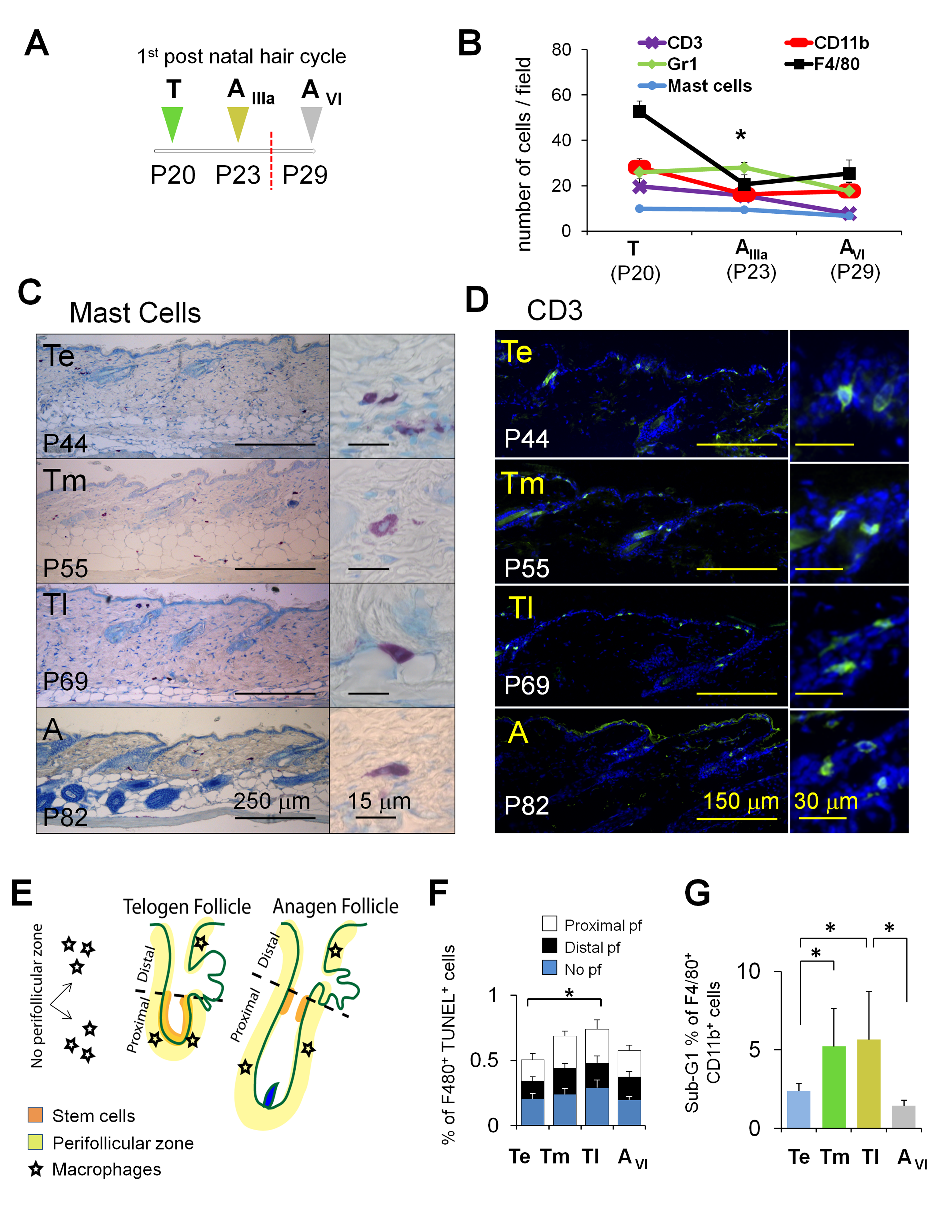

Supplement: Figure S1 — Skin-resident macrophages decrease in number before the onset of the first anagen. (A) Backskin samples were isolated from three different stages: P20, telogen (T); P23, early anagen (Ae), and P29, late anagen (Al). (B) Histograms show the fluctuations in number of different immune cell types analyzed by immunofluorescence. Each histogram point represents the mean value of positive cells per 10× magnification field. 10 fields/section/mouse were analyzed; n = 4. *p≤0.05. (C) Histological analysis of the expression of Toludine blue positive mast cells in the backskin at Te, Tm, Tl, and A stages. The boxed areas are shown at higher magnification in the right panels. (D) Immunofluorescence analysis of CD3 (green) counterstained with DAPI (blue) in the backskin at the specified HF stages; *p≤0.05. The boxed areas are shown at higher magnification in the right panels. (E) Diagram showing the perifollicular (proximal and distal) and no perifollicular regions used to assess the distribution of macrophages. (F) Histogram shows the percent of TUNEL+F4/80+ cells in mouse backskin at different stages; n = 3. The gating strategy is shown in Figure S3 A. (G) Histogram shows the percent of Sub-G1 DNA fragmentation of F4/80+CD11b+ sorted cells from skin at different stages; n = 3. The gating strategy is shown in Figure S11A. All data used to generate the histograms can be found in Data S1. (TIF) [file pbio.1002002.s001.tif]

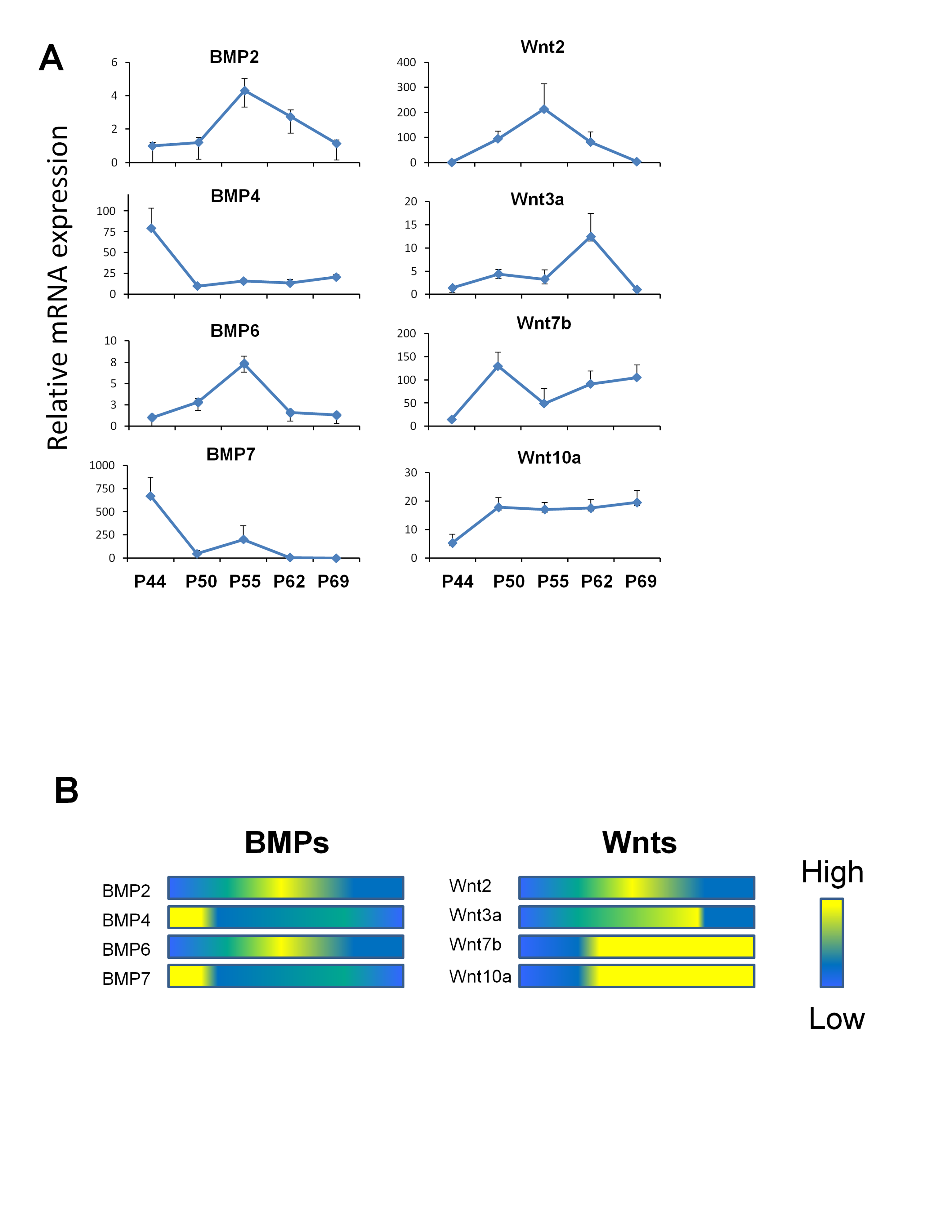

Supplement: Figure S2 — BMP/Wnt mRNA fluctuation at different telogenic stages. (A) Graphs represent the relative mRNA expression levels of BMPs and Wnts in total skin at different telogenic stages. (B) Graphical representation of the fluctuation of mRNA levels. All data used to generate the histograms can be found in Data S1. (TIF) [file pbio.1002002.s002.tif]

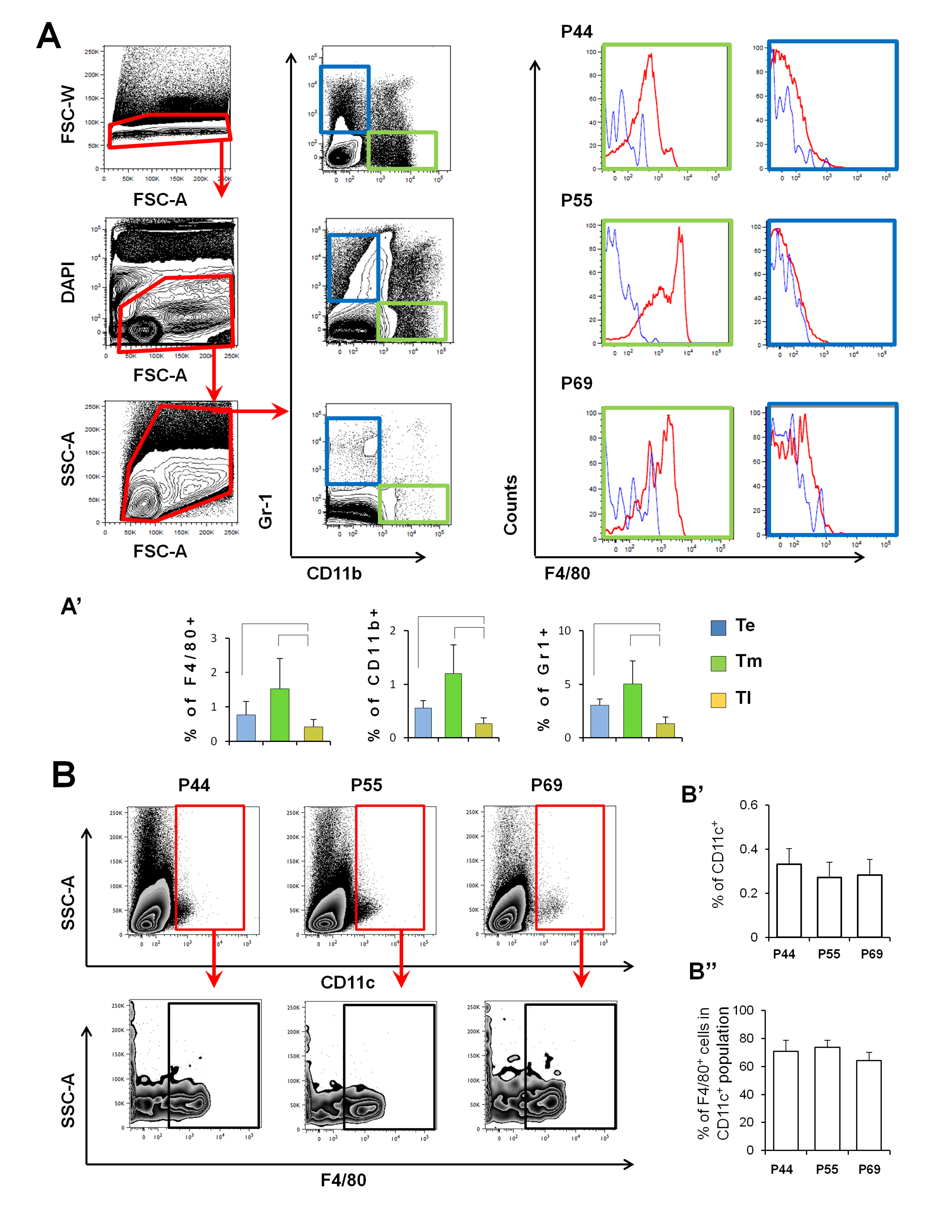

Supplement: Figure S3 — FACS analyses of macrophage populations in skin samples at different telogenic stages. (A) Gating strategy of single cell suspensions of total skin. Cells were analyzed by FACS and sorted by the differential expression of F4/80 on CD11b−Gr1+ and CD11b+Gr1− populations at different time points. (A′) Histograms represent the percent of single F4/80+,CD11b+,Gr1+ positive cells in the total skin at different telogenic stages. (B) Single cell suspensions were analyzed for the co-expression of CD11c and F4/80. (B′) Quantification of CD11c+ single cells and (B″) double positive CD11c+F4/80+ are shown in the histogram at different time points; n = 4. All data used to generate the histograms can be found in Data S1. The gating strategy is shown in Figure S11C. (TIF) [file pbio.1002002.s003.tif]

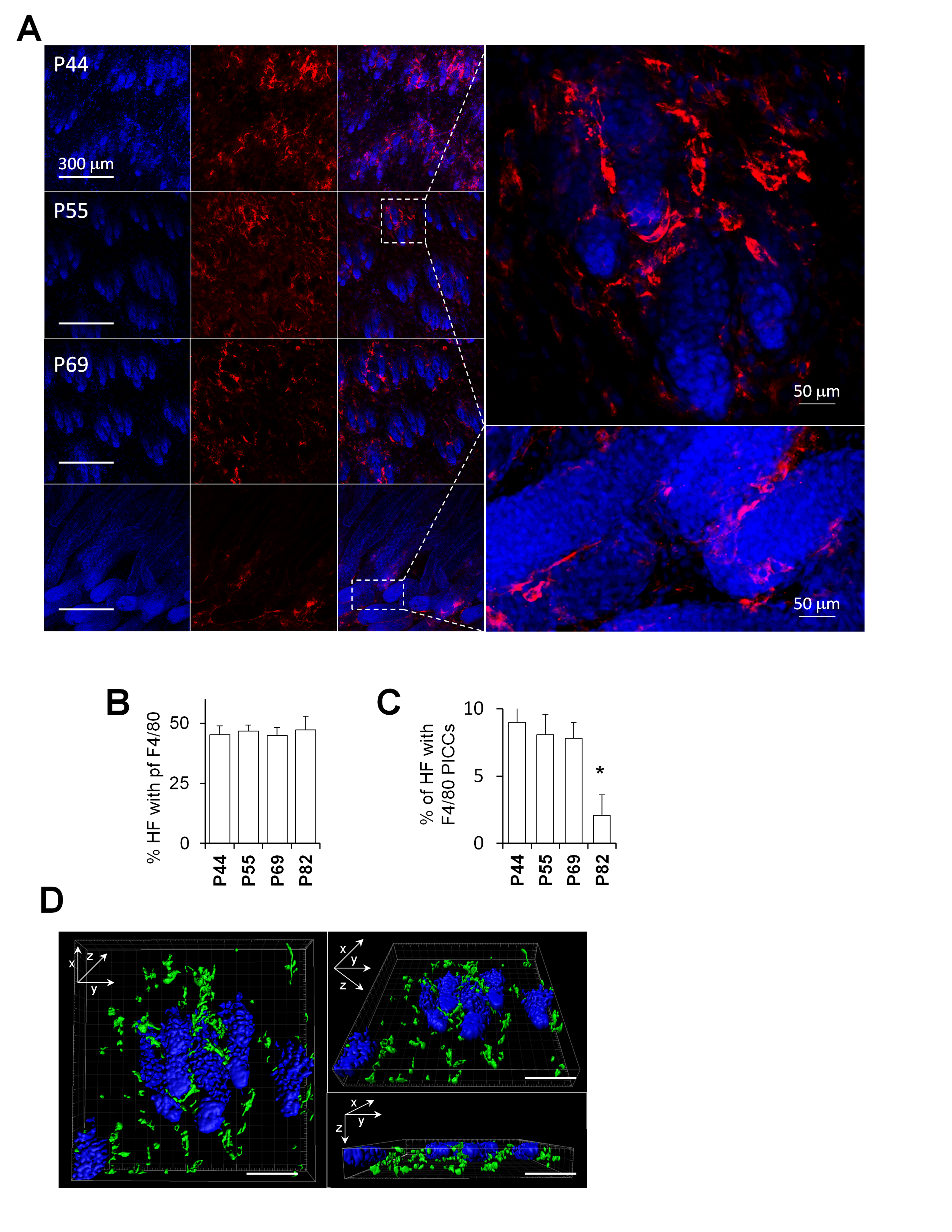

Supplement: Figure S4 — HFs exhibit perifollicular macrophages. (A) Immunofluorescence staining for F4/80+ cells (red) in whole mount skin preparations shown at different telogenic stages. (B) The histogram represents the percent of HFs exhibiting perifollicular macrophages. 200 HFs/mouse; n = 3. (C) The histogram represents the percent of HFs exhibiting PICCs clusters at different telogenic stages. 200 HFs/mouse; n = 3. (D) 3-D whole mount reconstruction of skin showing the distribution of F4/80+ cells, obtained using the Imaris software. *p≤0.05. All data used to generate the histograms can be found in Data S1. (TIF) [file pbio.1002002.s004.tif]

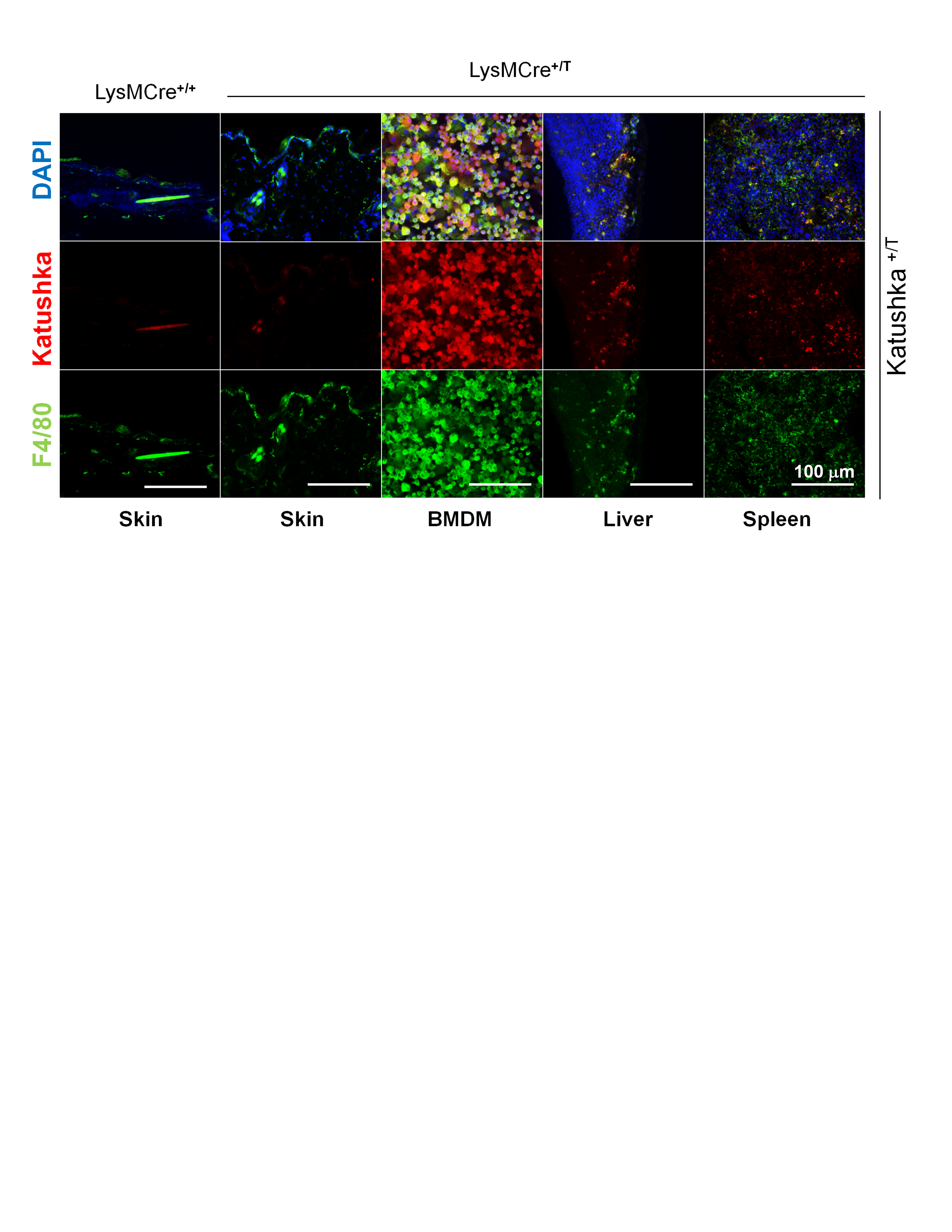

Supplement: Figure S5 — Skin resident macrophages do not present LysM-dependent expression of Cre under steady state conditions. Immunofluorescence analyses of backskin, cytospin of BMDM, liver and spleen derived from LysMCre+/T, iDTRKI/KI, and control LysMCre+/+, iDTR KI/KI mice under the background of the red fluorescent Katushka mice. F480+ (green), Katushka (red), DAPI (blue); n = 4 mice. (TIF) [file pbio.1002002.s005.tif]

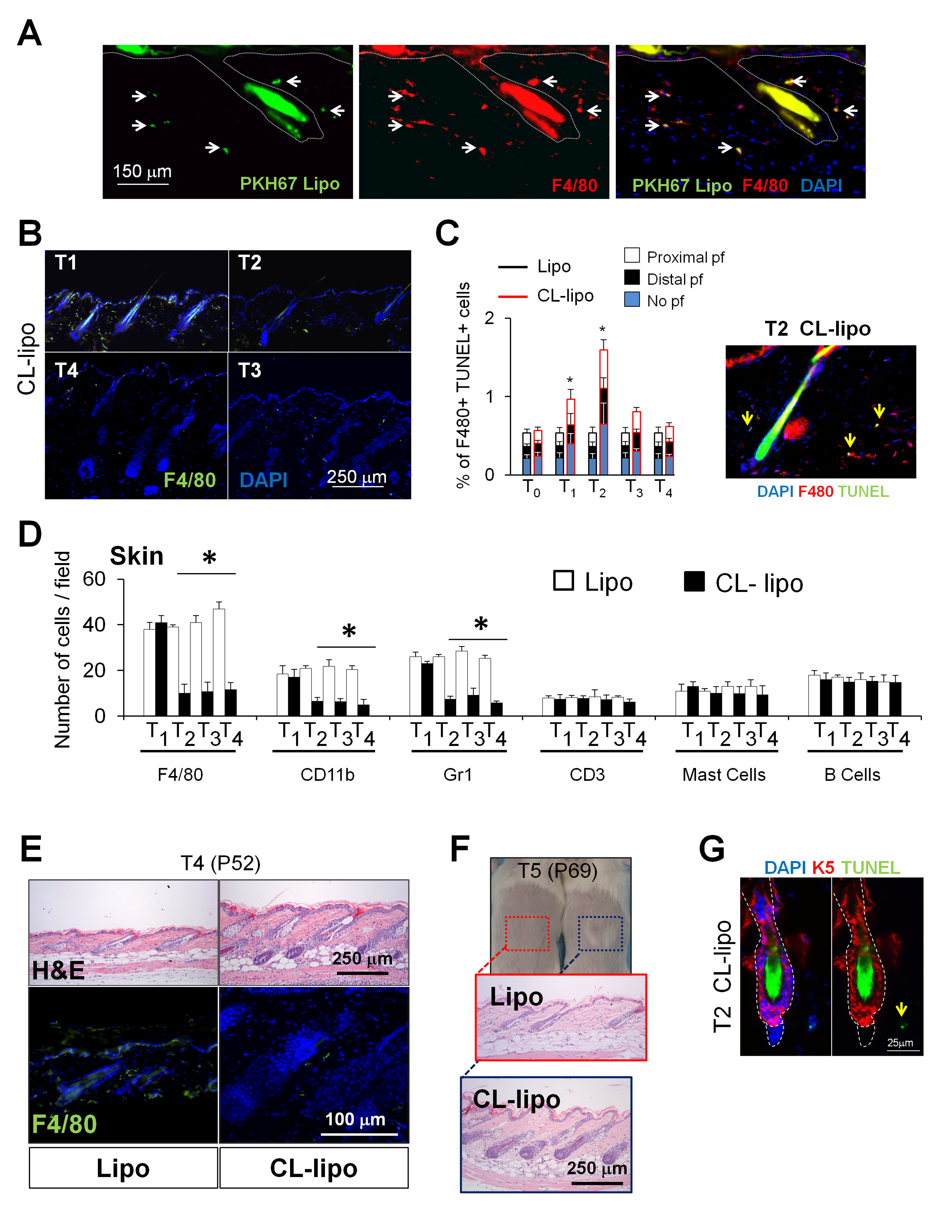

Supplement: Figure S6 — Subcutaneous administration of clodronate liposomes does not alter the number of other inflammatory cells in skin, and also is able to induce precocious HF growth at early telogen in FVB/N mice. (A) The specific uptake of liposomes by macrophages was analyzed by co-immunofluorescence analysis of F4/80+ cells (red) and the detection of the liposomal PKH67 label (green) in skin sections after the injection of PKH67-liposomes. Arrows indicate double labeling; n = 2. (B) Immunofluorescence of F4/80+ in backskin section of mice treated with CL-lipo and Lipo controls and collected at different time points; n = 4. (C) Left. Histogram shows the percent and the distribution of TUNEL+F4/80+ cells in the backskin of mice treated with CL-lipo and Lipo and analyzed at different time points; n = 3. Right. TUNEL and F4/80 immunofluorescence analyses in T2 backskin samples of mice treated with CL-lipo; n = 3. (D) Histogram shows the number of inflammatory cells present in skin sections after treatment with CL-lipo and Lipo controls, detected by immunofluorescence or histology techniques; n = 4. (E) FVB/N mice were injected in the backskin at T0 for two alternated days with CL-lipo. Samples were collected for analyses at T4 (P52). Hematoxylin–eosin staining of backskin samples isolated after treatment with CL-lipo and Lipo controls. Bar = 250 µm; n = 2. (F) Appearance of the hair coat at T5 (P69) in FVB/N mice, after shaving and treatment with CL-lipo and Lipo controls at T0 (P44). Bar = 250 µm; n = 2. (G) TUNEL and K5 immunofluorescence analyses in T2 backskin samples of mice treated with CL-lipo; n = 3. All data used to generate the histograms can be found in Data S1. (TIF) [file pbio.1002002.s006.tif]

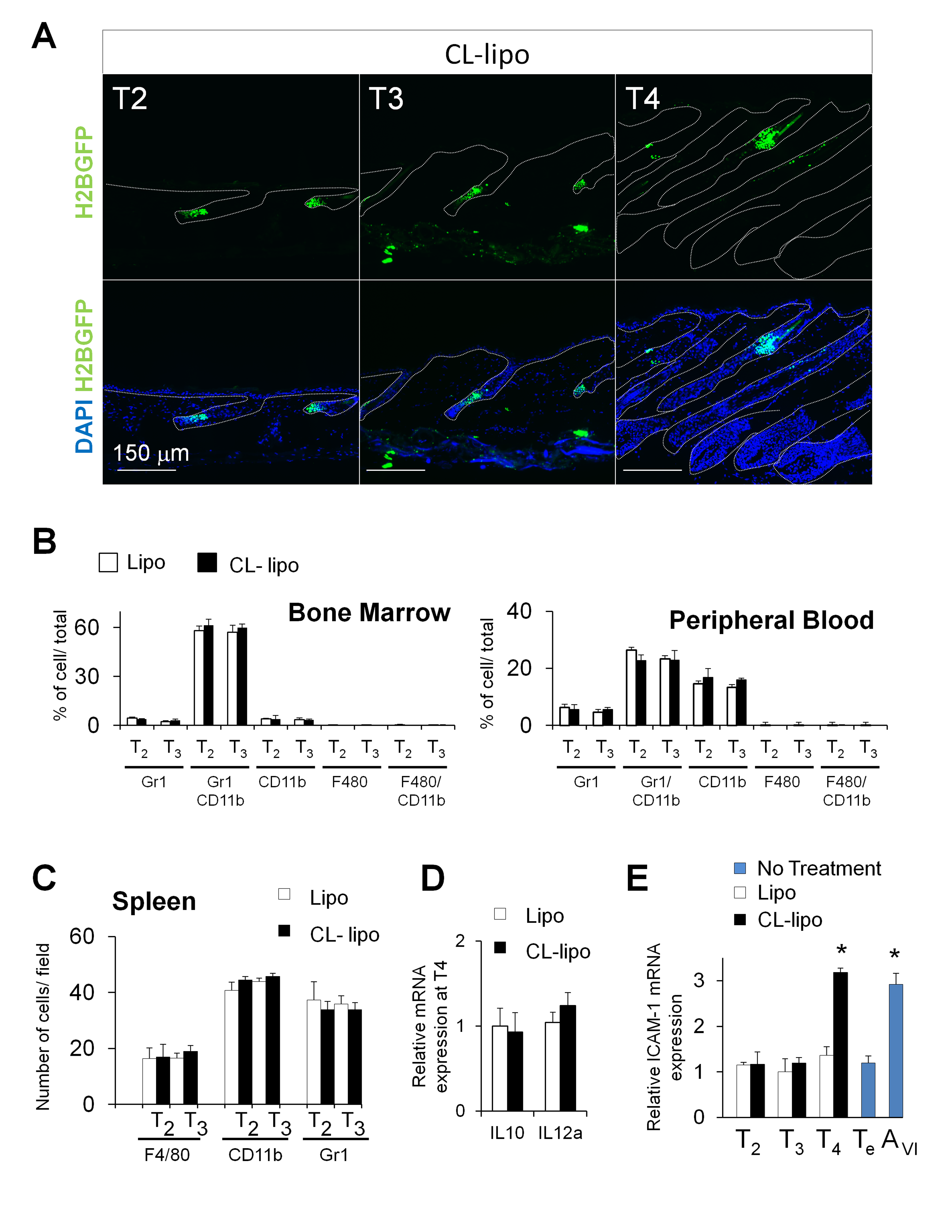

Supplement: Figure S7 — Subcutaneous administration of clodronate liposomes does not alter macrophage number in the spleen, blood, and bone marrow, nor does it induce skin inflammation. (A) Representative backskin sections of K5tTA-pTREH2B-GFP mice, subjected to a pulse-chase treatment with doxycycline, followed by treatment at P56 for two alternated days with CL-lipo or Lipo controls. (B) Histograms show the number of F4/80, CD11b, and Gr1 positive cells in the bone marrow and peripheral blood detected by FACS, after subcutaneous treatment with CL-lipo and Lipo controls; n = 3. The gating strategy is shown in Figure S11D and S11E. (C) Histograms show the number of F4/80, CD11b, and Gr1 positive cells in the spleen detected by IF, after subcutaneous treatment with CL-lipo and Lipo controls; n = 3. (D) Histograms represent the relative mRNA expression levels of IL10 and IL12 at T4 in Lipo versus CL-lipo treated backskin; n = 3. (E) Histograms represent the relative ICAM1 mRNA expression levels at T2, T3, and T4 in Lipo versus CL-lipo treated backskin, and untreated Te and AVI; n = 3. *p≤0.05. All data used to generate the histograms can be found in Data S1. (TIF) [file pbio.1002002.s007.tif]

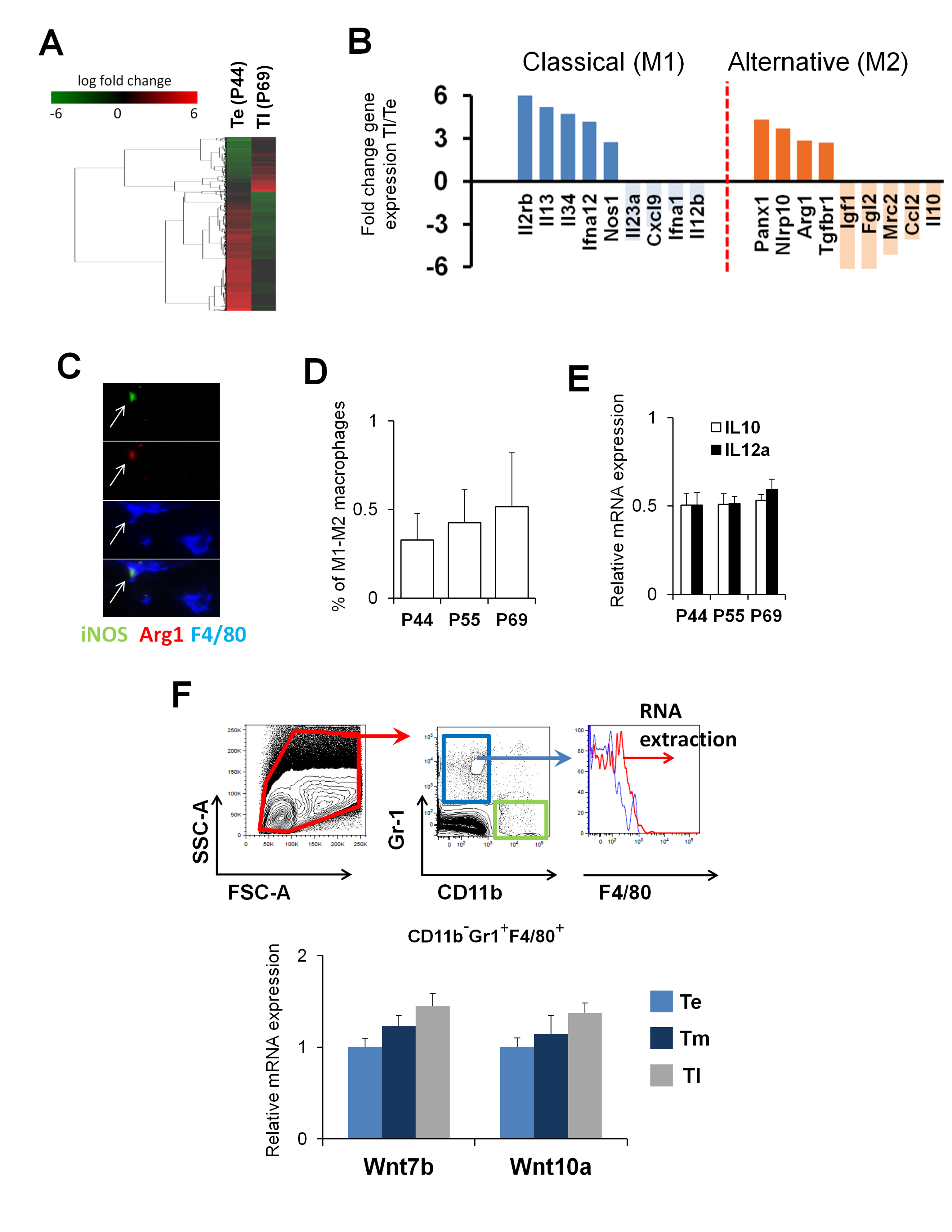

Supplement: Figure S8 — Macrophage gene expression during the Te to Tl transition. (A) Unsupervised clustering heat map showing genes up- and downregulated in F4/80+CD11b+ cells isolated from backskin of mice at Te and Tl. (B) Histograms show a shortlist of classical or alternative macrophage associated genes that were found up- or downregulated in FACS-isolated CD11b+Gr1−F4/80+ mature macrophages, using microarray analyses. The gating strategy is shown in Figure S3A. (C) Immunofluorescence analysis of skin sections of iNOS (M1), Arg1 (M2), and F4/80. (D) Histograms represent the percentage of Arg1/iNOS double positive F4/80 cells in skin at different telogenic stages. (E) Histograms show the relative mRNA expression of IL10 and IL12a in whole backskin of mice and time point indicated; n = 3. (F) Relative mRNA expression of Wnt7b and Wnt10a in FACS-isolated F4/80+ cells present in the CD11b−Gr1+ population at Te, Tm, and Tl; n = 3. The gating strategy is shown in Figure S3 A. All data used to generate the histograms can be found in Data S1. (TIF) [file pbio.1002002.s008.tif]

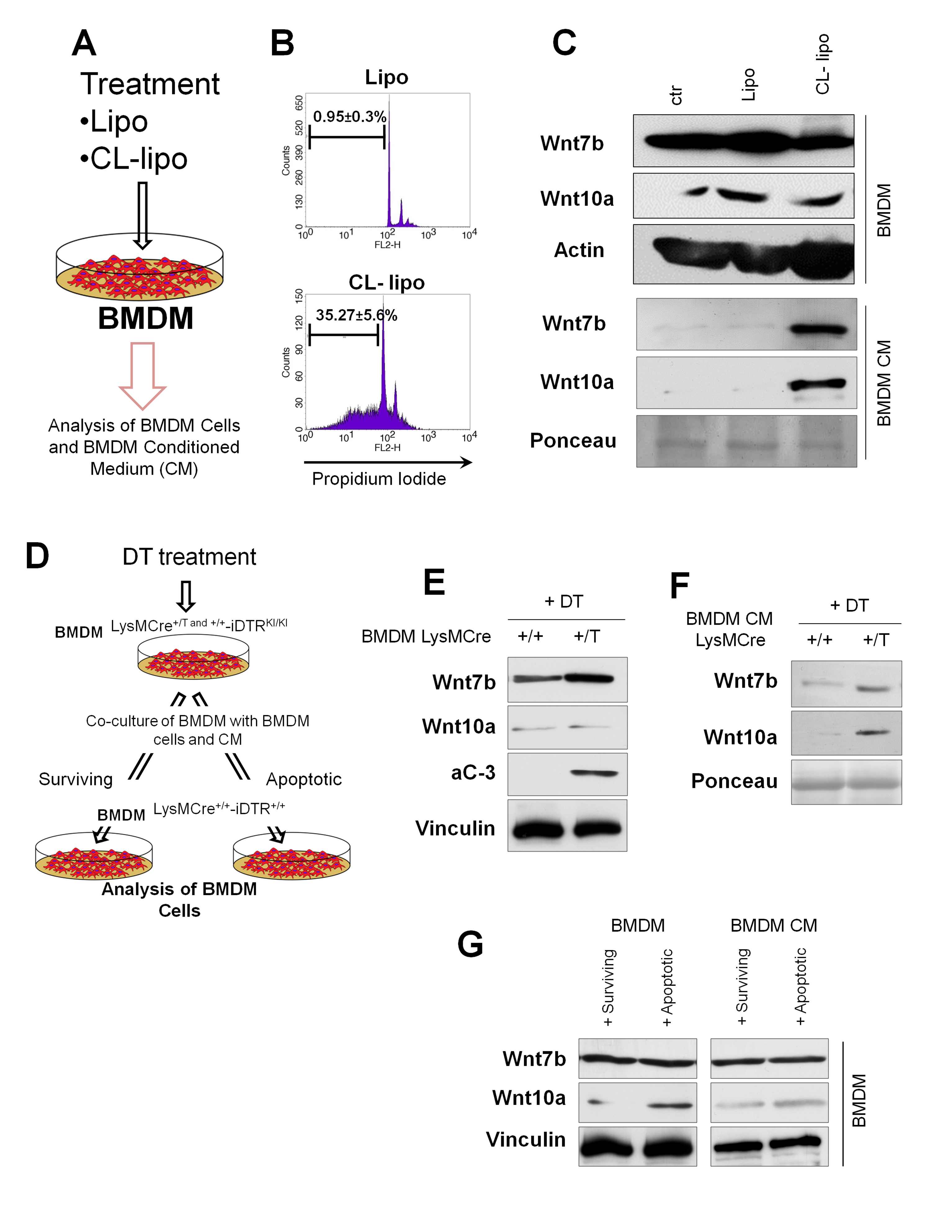

Supplement: Figure S9 — Treatment of bone marrow differentiated macrophages with clodronate-liposomes releases Wnt7b and Wnt10a. (A) Scheme illustrating the treatment of BMDM with either CL-lipo or Lipo controls, before harvesting treated cells or their conditioned medium (BMDM CM). (B) FACS analysis of the sub-G1 DNA content of BMDM treated with CL-lipo and Lipo controls; n = 3. Bars indicate the percentage of cell death. The gating strategy is shown in Figure S11 F. (C) Immunoblot analysis of Wnt7b and Wnt10a expression in both BMDM total cell lysates and BMDM CM treated with CL-lipo and Lipo controls. (D) Scheme illustrating the experimental approach used to explore the effect of apoptosis in the expression of Wnts. BMDM derived from LysMCre+/T-iDTRKI/KI mice or control LysMCre+/+iDTRKI/KI were treated with diphteria toxin (DT). Floating apoptotic (LysMCre+/T-iDTRKI/KI+DT) and alive attached (LysMCre+/+iDTRKI/KI+DT) macrophages were collected, and used to treat control BMDM in a 1∶1 ratio. (E) Immunoblot analysis of Wnt7b and Wnt10a and active caspase-3 (AC3) expression in BMDM and CM isolated from both LysMCre+/T-iDTRKI/KI and control LysMCre+/+iDTRKI/KI mice treated with DT; n = 3. (F) Immunoblot analysis of Wnt7b and Wnt10a expression in BMDM and CM isolated from LysMCre+/+iDTRKI/KI mice treated with BMDM LysMCre+/T-iDTRKI/KI and control LysMCre+/+iDTRKI/KI mice treated with DT; n = 3. n refers to number of experimental replicates. (G) Immunoblot analysis of Wnt7b and Wnt10a expression in fresh BMDM treated with surviving LysMCre+/+iDTRKI/KI cells or apoptotic LysMCre+/T-iDTRKI/KI cells, or with their respective CM; n = 3. n refers to number of experimental replicates. All data used to generate the histograms can be found in . (TIF) [file pbio.1002002.s009.tif]

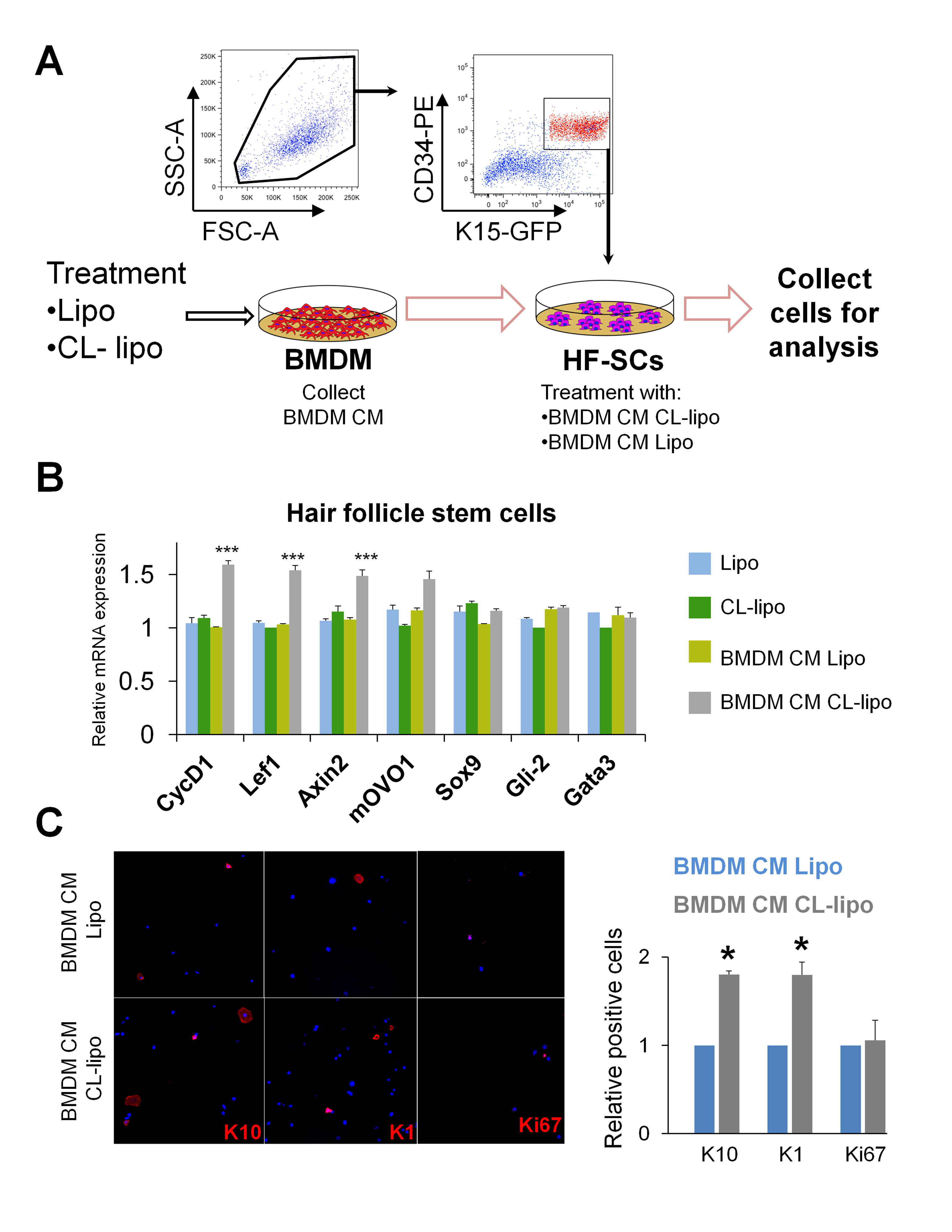

Supplement: Figure S10 — Macrophage derived soluble factors promote in vitro HF-SC activation and differentiation. (A) Scheme representing the protocol used to stimulate HF-SCs with macrophage CM. BMDM cells were treated with either CL-lipo or Lipo controls. The media was collected and used to treat FACS-isolated GFP+, CD34+ HF-SCs growing in culture. The gating strategy is shown in Figure S11G. (B) Relative mRNA expression of HF-SCs treated with Lipo control, CL-lipo, or the BMDM CM of cells treated with Lipo and CL-lipo; n = 9. n refers to number of experimental replicates. (C) Immunofluorescence analysis of K1, K10, and Ki67 (red) in HF-SCs treated with CL-lipo BMDM CM when compared to controls. The histogram shows the quantification of positive cells; n = 3. *p≤0.05; ***p<0.0005. All data used to generate the histograms can be found in Data S1. (TIF) [file pbio.1002002.s010.tif]

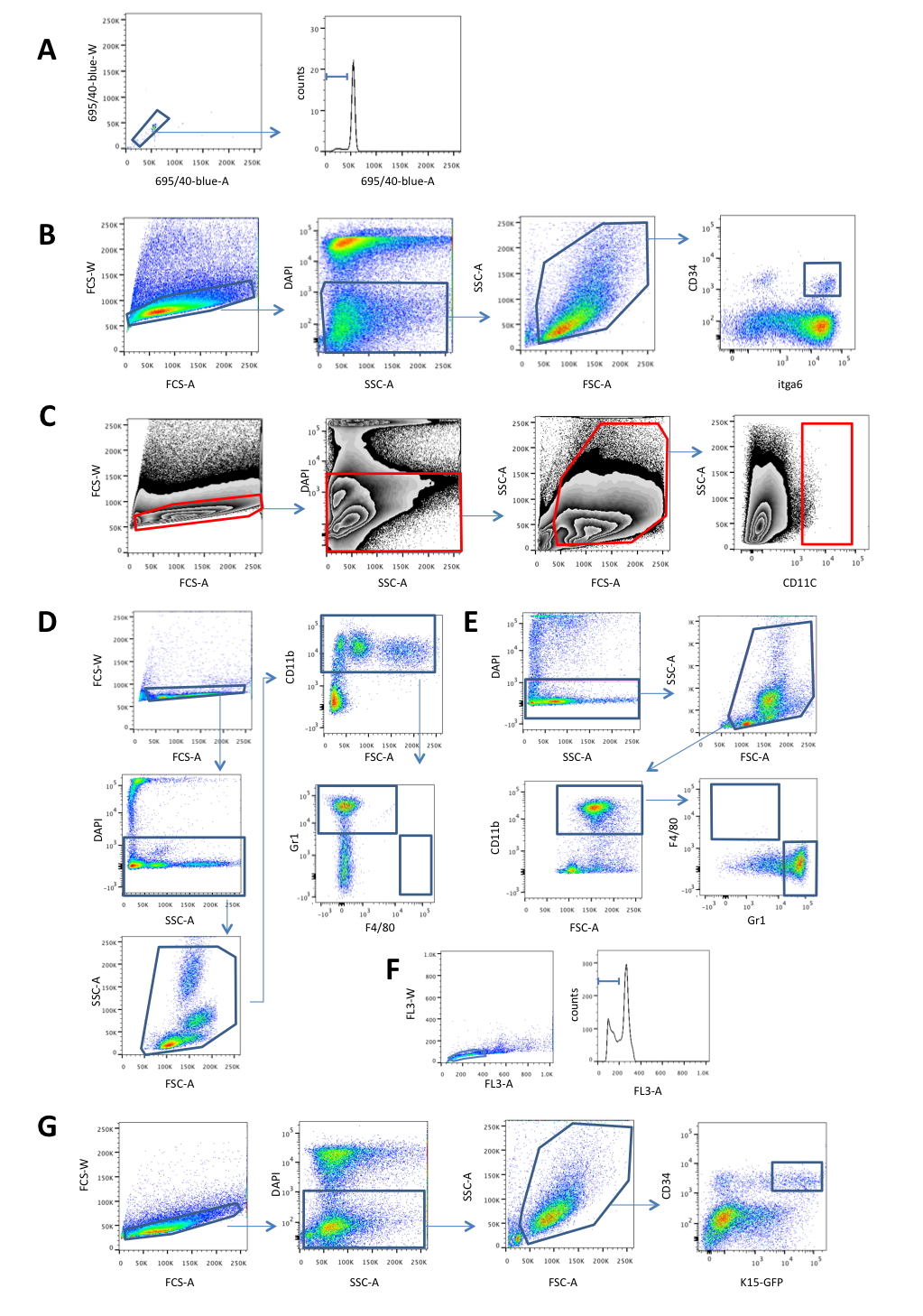

Supplement: Figure S11 — Gating strategy of the flow cytometry analyses presented in this study. The gating strategy is presented in Figures 1F, 4B, S1G, S3B, S7B, S9B, and S10A. (TIF) [file pbio.1002002.s011.tif]
