# Supplementary material for: Macrophages Contribute to the Cyclic Activation of Adult Hair Follicle Stem Cells
Source: PLoS Biol. 2014 Dec 23;12(12):e1002002. doi: 10.1371/journal.pbio.1002002 (PMC4275176; doi:10.1371/journal.pbio.1002002)
Supplement: Text S1 — Supplementary materials and methods. (DOC) [file pbio.1002002.s013.doc]

**SUPPORTING INFORMATION**

**Cell Culture**

Freshly isolated bone marrows of CD-1 mice and LysMCre^+/T^ iDTR^KI/KI^ (Jackson Lab) were used to generate crude populations of bone-marrow-derived-macrophages (BMDM) *in vitro*, as previously described [1]. Briefly, bone marrow cells were cultured for 7 days in DMEM medium supplemented with 10% FBS, 100 U/ml penicillin/streptomycin, and 10 ng/ml recombinant mouse M-CSF (PeproTech EC). For coculture experiments, BMDM cells were washed in PBS, and grown in 3:1 DMEM/F12 media supplemented with 5% FBS, 100 U/ml penicillin/streptomycin and 0.3 mM Ca^2+^. For the generation of HF-SC cultures, K15-GFP, CD34^+^ FACS sorted cells were plated on fibronectin coated dishes and grown in 3:1 DMEM/F12 media supplemented with 15% FBS, 100 U/ml penicillin/streptomycin and 0.3 mM Ca^2+^. HF-SCs were incubated for 48 h in conditioned media of BMDM cultures treated with clodronate or control liposomes. For alive/death BMDM experiments, BMDM were treated for 24h with LysMCre^+/T^iDTR^KI/KI^ or control LysMCre^+/+^iDTR^KI/KI^ BMDM pretreated overnight with DT (1μg/ml).

**Western Blotting**

Cells in culture were lysed in a buffer containing 50mM Tris pH7.4, 150 mM NaCl, 5 mM EDTA, 5 mM EGTA, 1% Triton X-100, 1% NP40, 2 mM sodium orthovanadate, 1 mM PMSF and protease inhibitors. BMDM conditioned media were concentrated 15 times using Amicon ultra centrifugal filters (Millipore). Protein lysates or conditioned media were separated by electrophoresis in SDS-polyacrylamide gels and transferred onto nitrocellulose membranes. Blots were blocked and incubated with Wnt7b (mouse mAb, MAB3460 R&D Systems) and Wnt10a (rabbit pAb, AB106522 Abcam and Rat pAb, MAB34691, R&D), β-actin (mouse Ab, Sigma Aldrich A5441), vinculin (mouse Ab, sigma Aldrich V9131) and active Caspase 3 (rabbit pAb, Cell Signaling, 9661) primary antibodies. After incubation with secondary horseradish peroxidase-conjugated antibodies, the signals were detected with the ECL Western Blotting detection system (Amersham, GE Healthcare, UK).

**Immunohistochemistry, immunofluorescence, whole mount and antibodies**

Skins were embedded in OCT compound and frozen, or fixed overnight with buffered 4% PFA at 4°C and embedded in paraffin. 7 μm sections of frozen skin were fixed in 4% PFA and immunostained with different antibodies according to manufacturer’s instructions. 3 μm paraffin-embedded skin sections were stained with hematoxylin and eosin for histological analysis or processed for immunostaining. Slides were blocked with PBS, 0.3% Triton X-100, 1% BSA, 5% normal goat serum, 5% normal donkey serum, or MOM Basic kit (Vector Labs). Images were acquired using an Eclipse 90i fluorescent microscope (Nikon Instruments) adapted with a DS-QiMC digital camera, and the NIS-elements acquisition software (Nikon Instruments). Images were processed with the Photoshop CS4 software (Adobe Systems), and quantifications were performed using the NIS or the ImageJ software (NIH).

Histomorphometric analyses were carried out as previously described[2,3]. Briefly, HF stages throughout the hair cycle were evaluated respecting comprehensive guide of for the accurate classification of murine HF[2]. Perifollicular F4/80+ positive cell counts were evaluated in 31 μm wide dermal strip compartments around the HF as previously reported[2]. HF counting was assed starting at midline, and spaced by lateral interval of 90 μm to exclude double HF counting. Positive cells were counted in 10x magnification fields and normalized to total skin area.

For whole mount, skin was isolated from mice at specific time points, fixed overnight in PFA 4%. Tissues were blocked and permeabilized in PBS, 0.5% NGS, 0.5% NDS, 1%BSA, 1%Tween, 1%Triton. Stainings were conducted O.N. at 4C. Images were captured using a Leica TCS SP5 (AOBS) confocal microscope. 3D reconstruction were produced with Imaris software (Bitplane).

The following primary antibodies were used: β-catenin (β-cat, mAb Clone 15B8, C7207 Sigma), CD3ε (mouse mAb Clone APA1/1, 05-785 Millipore), CD11b (mouse mAb Clone ICRF44, 555386 BD Pharmigen), CD11c (hamster mAb Clone G235-2356, 553954 BD Pharmigen), CD34 (rat mAb Clone RAM34, 553731 BD Pharmigen), F4/80 (rat mAb Clone CI:A3-1, MCA497G Serotec), Gr1/Ly6G (rat mAb clone RB6-8C5, MAB1037 R&D Systems), Keratin-5 (K5, rabbit pAb, PRB-160P Covance), Keratin-6 (K6, rabbit pAb, PRB-169P Covance), Ki67 (rabbit pAb, NB500-170 Novus Biological), P-cadherin (P-cad, rat mAb Clone PCD1, 13-2000Z Zymed Laboratories), Tenascin C (TenC, rabbit pAb, AB19011 Millipore), TurboRFP (Katushka, rabbit pAb, AB233 Evrogen), iNOS (rabbit pAb, 610333 Becton Dickinson) and Arg1 (goat pAb, SC-18534 SantaCruz).

**RNA isolation and Real Time-PCR**

FACS isolated skin-resident macrophages were pooled from 4 littermate mice per point. Total RNA was isolated from cells and tissues using TRIZOL (Invitrogen), and purified using the Absolutely RNA reverse transcription system (Stratagene). After DNAse treatment, 2 μg were used for cDNA synthesis using the Ready to Go You Prime It First-Strand beads and random primers (GE Healthcare). RT-PCR reactions were conducted using the GoTaq qPCR Master Mix (Promega) and a MasterCycler Ep-Realplex thermal cycler (Eppendorf). The following settings were used: 2 min at 95° C for initial denaturing, 35 cycles of 15 s at 95°C denaturing, 40 s at 57°C annealing, and 45 s at 72°C extension. The expression levels of PCR products were normalized according to GAPDH or β-actin. The relative expression of mRNAs between samples and controls was calculated using the comparative ddCt method. The primers used to perform the PCR reactions are indicated below:

Actin Fw 5’ cacagctgagagggaaatcg 3’ Rv 5’ agtttcatggatgccacagg 3’

AE15 Fw 5’ gagctaagacgcgagcaaga 3’ Rv 5’ ccacctctgggtaatgctct 3’

Axin2 Fw 5’ gagagtgagcggcagagc 3’ Rv 5’ cggctgactcgttctcct 3’

BMP2 Fw 5’ agatctgtaccgcaggcact 3’ Rv 5’ gttcctccacggcttcttc 3’

BMP4 Fw 5’ gaggagtttccatcacgaaga 3’ Rv 5’ gctctgccgaggagatca 3’

BMP6 Fw 5’ actgactagcgcgcagga Rv 5’ tgtggggagaactccttgtc

BMP7 Fw 5’ cgagaccttccagatcacagt Rv 5’ cagcaagaagaggtccgact

CyclinD1 Fw 5’ gcgtaccctgacaccaatctc 3’ Rv 5’ ctcctcttgcattctgctc 3’

Gapdh Fw 5’ cctggagaaacctgccaagtatg 3’ Rv 5’ agagtgggagttgctgttgaagtc 3’

Gata3 Fw 5’ ttatcaagcccaagcgaag 3’ Rv 5’ tggtggtggtctgacagttc 3’

Gli2 Fw 5’ gcagactgcaccaaggagta 3’ Rv 5’ cgtggatgtgttcattgttga 3’

ICAM1 Fw 5’ cttccagctaccatcccaaa Rv 5’ cttcagaggcaggaaacagg

K6 Fw 5’ agtttgcctccttcatcgac 3’ Rv 5’ tgctcaaacataggctccag 3’

K6irs Fw 5’ tggacgcagcttacatgaac 3’ Rv 5’ tctctgccttgctcttcaca 3’

K34 Fw 5’ gtggagtctctgagggagga 3’ Rv 5’ cgcagggtgttgacttcc 3’

Lef1 Fw 5’ tcctgaaatccccaccttct 3’ Rv 5’ tgggataaacaggctgacct 3’

mOvo1 Fw 5’ gcgagatctacgtgccagtc 3’ Rv 5’ caccgatgcctctggttc 3’

Sox9 Fw 5’ tgcccatgcccgtgcgtcaa 3’ Rv 5’ cgctccgcctcctccacgaagggtct 3’

Wnt2 Fw 5’ cctgatgaaccttcacaacaac Rv 5’ tcttgtttcaagaagcgctttac

Wnt3a Fw 5’ ctcgctggctacccaattt Rv 5’ gaggccagagatgtgtactgc

Wnt7b Fw 5’ gcgtcctctacgtgaagctc 3’ Rv 5’ tcttgttgcagatgatgttgg 3’

Wnt10a Fw 5’ tcctattcttcctactactact 3’ Rv 5’ aaaatatcattaaatact 3’

IL10 Fw 5’ cagagccacatgctcctaga Rv 5’ gtccagctggtcctttgttt

IL12a Fw 5’ ccaggtgtcttagccagtcc Rv 5’ gcagtgcaggaataatgtttca

**References**

1. Weischenfeldt J, Porse B (2008) Bone Marrow-Derived Macrophages (BMM): Isolation and Applications. CSH Protoc 2008: pdb prot5080.

2. Paus R, van der Veen C, Eichmuller S, Kopp T, Hagen E, et al. (1998) Generation and cyclic remodeling of the hair follicle immune system in mice. J Invest Dermatol 111: 7-18.

3. Muller-Rover S, Handjiski B, van der Veen C, Eichmuller S, Foitzik K, et al. (2001) A comprehensive guide for the accurate classification of murine hair follicles in distinct hair cycle stages. J Invest Dermatol 117: 3-15.
